# Chromatin-bound U2AF2 splicing factor ensures exon inclusion

**DOI:** 10.1016/j.molcel.2025.04.013

**Published:** 2025-05-01

**Authors:** Weifang Wu, Kami Ahmad, Steven Henikoff

**Affiliations:** 1 Basic Sciences Division, Fred Hutchinson Cancer Center, Seattle, WA 98109, USA; 2 Howard Hughes Medical Institute, Chevy Chase, MD 20815, USA; 3 Lead contact

## Abstract

Most mRNA splicing occurs co-transcriptionally, but it is unclear how splicing factors accurately select exons for inclusion. Using CUT&RUN profiling in K562 cells, we demonstrate that three splicing factors—SF3B1, U2AF1, and U2AF2—bind near active promoters of intron-containing and intronless genes, implying their association with the general transcriptional machinery. RNase A treatment reduces factor binding at promoters, indicating that these proteins interact with nascent transcripts. Strikingly, the U2AF2 protein also accumulates throughout intron-containing gene bodies and requires histone H3-lysine36 trimethylation but not nascent transcripts or persistent RNA polymerase II. Chromatin-bound U2AF2 preferentially binds to exons of highly expressed, exon-dense genes, with greater occupancy at exons skipped after U2AF2 knockdown, suggesting that U2AF2 enhances exon selection accuracy. U2AF2-targeted genes include those encoding splicing factors, where it improves splicing accuracy and efficiency. Our findings provide a mechanistic basis for the homeostatic regulation of efficient co-transcriptional splicing by chromatin-bound U2AF2.

## In brief

Wu et al. reveal that the splicing factor U2AF2 binds chromatin independently of RNA to guide accurate exon selection during transcription. Their findings uncover a chromatin-based mechanism that enhances splicing efficiency and ensures proper expression of genes essential for RNA processing.

## Graphical Abstract

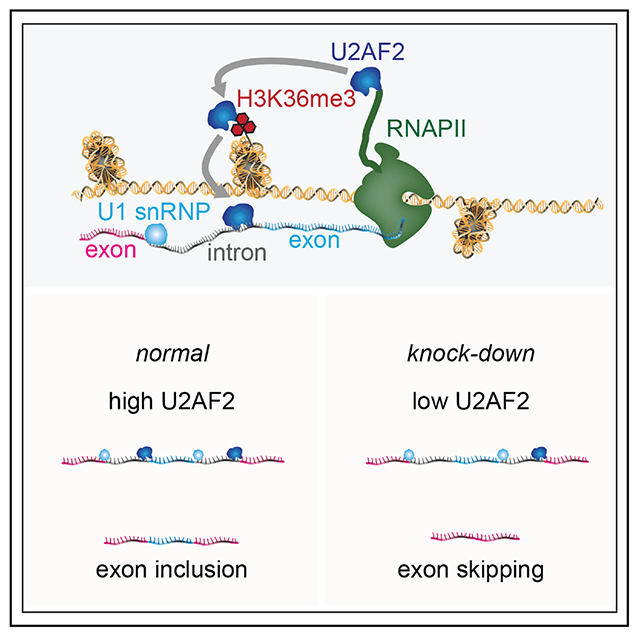

## INTRODUCTION

To form mature mRNAs, most eukaryotic precursor mRNAs (pre-mRNAs) require intron removal and exon connection. This splicing reaction is catalyzed by the spliceosome, a protein complex that includes U1, U2, and U4/U6·U5 small nuclear ribonucleoproteins (snRNPs) and various splicing factor proteins.^1,2^ Exons are separated by an intron, where four main consensus sequences are identified from 5′ to 3′: the 5′ splice site, the branchpoint sequence, the polypyrimidine tract, and the 3′ splice site. Spliceosome recognition of exon-intron junctions begins with U1 snRNP binding to the 5′ splice site, U2 snRNP auxiliary factor 35 (U2AF1) recognizing the 3′ splice site, and U2 snRNP component SF3B1 binding the branchpoint.^1,3^ Since the branchpoint sequence is short and poorly conserved in metazoans, U2 snRNP auxiliary factor 65 (U2AF2) binds to the polypyrimidine tract to stabilize U2 snRNP’s association with the branchpoint.^1,4^ Subsequent recruitment of the preassembled U4/U6·U5 tri-snRNP and a series of conformational and compositional rearrangements activate the spliceosome for splicing reactions.^1,3^

Most splicing occurs co-transcriptionally, whereby introns are removed and exons are joined while the nascent pre-mRNA is still being synthesized by RNA polymerase II (RNAPII) as it transcribes the DNA template.^5,6^ Co-transcriptional splicing requires SF3B1, U2AF1, and U2AF2 to bind to pre-mRNA intron-exon junctions just after RNAPII transit. The consensus sequences in pre-mRNA regulate exon inclusion by influencing the binding of these splicing factors. Additional features within exons might further aid in their inclusion by these splicing factors, and these are on DNA that is densely packaged into chromatin.^7–10^ Exons in vertebrate protein-coding genes are typically uniform in length (~140–150 nt), similar to the 147-bp length of DNA that wraps around a nucleosome, whereas introns are longer and more variable.^11^ Exons are higher in guanine-cytosine (GC) content, nucleosome occupancy, and the active histone H3-lysine36 trimethylation (H3K36me3) mark compared with flanking introns.^7,12,13^ In this study, we demonstrate that the SF3B1, U2AF1, and U2AF2 splicing factors localize to active gene promoters in an RNA-dependent manner, implying that they bind to nascent transcripts. Notably, U2AF2 also binds to chromatin independently of RNA within the bodies of actively transcribed, exon-dense genes and remains bound to H3K36me3-marked chromatin even when RNAPII transcription is inhibited. Chromatin-bound U2AF2 enhances splicing accuracy and efficiency in highly expressed, exon-dense genes, including a network of splicing factor genes, suggesting that it acts as a homeostatic regulator to ensure efficient co-transcriptional splicing.

## RESULTS

### Splicing factors bind near the promoters of all active genes

**Figure 1.**
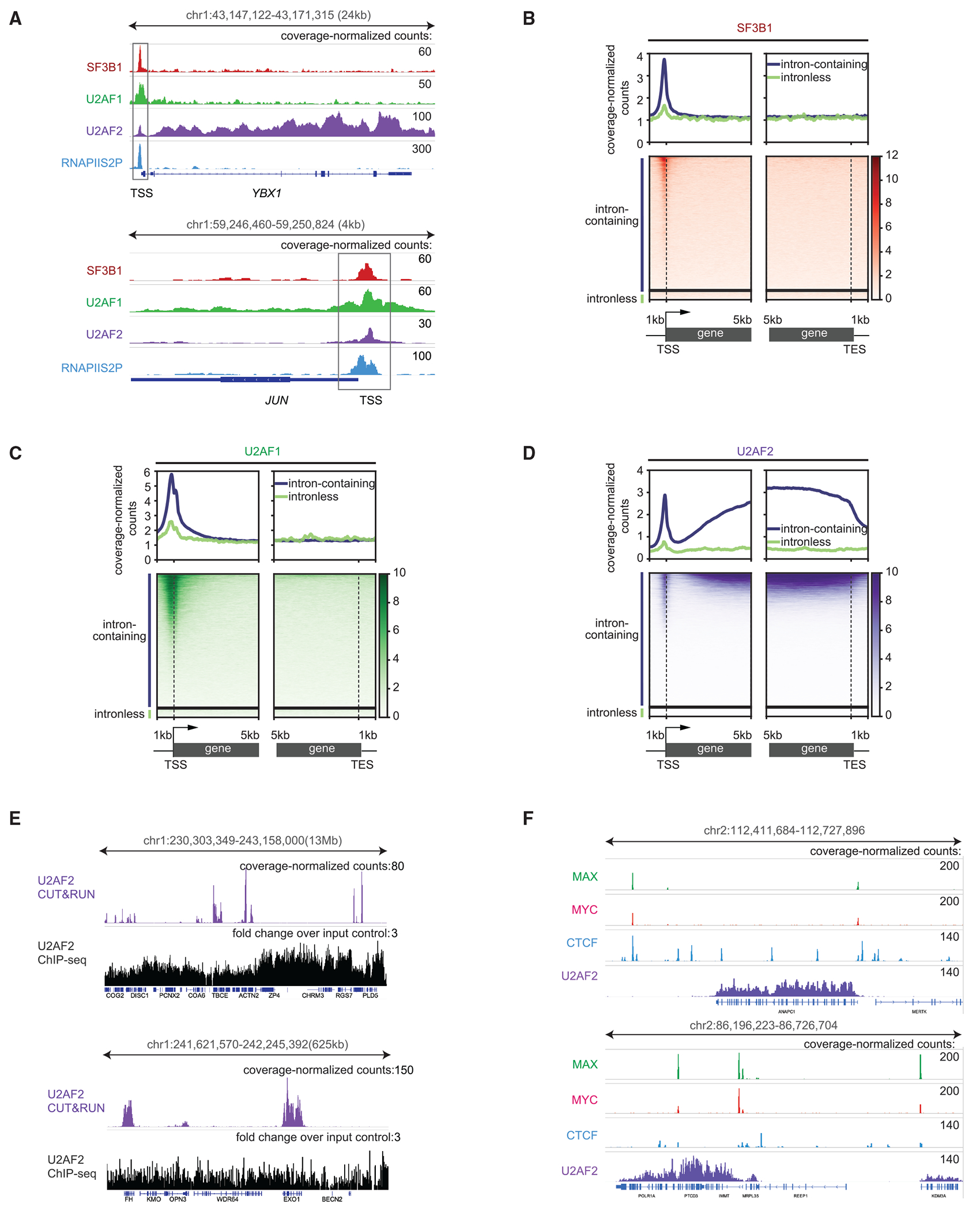
Splicing factors bind near the promoters of all active genes (A) Browser track showing SF3B1, U2AF1, U2AF2, and RNAPIIS2P. (B–D) Heatmaps (bottom) and average plots (top), aligned to the TSS or TES of 12,397 intron-containing and 793 intronless genes, displaying coverage-normalized counts for SF3B1 (B), U2AF1 (C), and U2AF2 (D). Each row in the heatmap represents one gene. See also Figures S1 and S2. (E) Browser track showing U2AF2 CUT&RUN and ChIP-seq signals. The *y* axis shows coverage-normalized counts for CUT&RUN and fold change over input for ChIP-seq. (F) Browser track showing CUT&RUN signals for CTCF, MYC, Max, and U2AF2.

Previous results have suggested that some splicing factors associate with RNAPII.^14,15^ Therefore, we aimed to determine whether SF3B1, U2AF1, and U2AF2 bind to chromatin. We used CUT&RUN (cleavage under targets and release using nuclease), a high-resolution chromatin profiling technique that maps protein epitopes *in situ*,^16^ to identify the genome-wide positions of splicing factors on chromatin in human K562 cells. We determined the locations of splicing factors on genes by aligning their coverage-normalized fragment counts around transcription start sites (TSSs) and around termination sites (TESs). Coverage-normalized counts are vital for identifying the chromatin enrichment signals of splicing factors. We observed SF3B1, U2AF1, and U2AF2 at TSSs of intron-containing and of intronless genes (Figures 1A–1D). Their levels at these TSSs positively correlated with each other (Figure S1A), indicating co-localization. Given that most splicing occurs co-transcriptionally, we compared the occupancy of SF3B1, U2AF1, and U2AF2 with that of elongating RNAPII phosphorylated on the serine-2 residue (RNAPIIS2P) at intron-containing and intronless gene TSSs. We observed that SF3B1, U2AF1, and U2AF2 levels positively correlated with RNAPIIS2P levels at these TSSs (Figure S1B). To test whether CUT&RUN can distinguish protein-DNA-binding sites from accessible DNA regions, we performed CUT&RUN on the NPAT nuclear factor, which binds exclusively to histone genes. We have previously used NPAT to distinguish specific binding from open chromatin, such as at active TSSs.^17,18^ We observed that NPAT CUT&RUN signals are undetectable at other active genes marked by RNAPIIS2P CUT&RUN signals (Figures S1C–S1E). These findings confirm that SF3B1, U2AF1, and U2AF2 bind to all active promoters.

As these splicing factors concentrate in nuclear speckles,^19–22^ we investigated whether splicing factor signals at TSSs result from genes co-localizing with nuclear speckles. The published SON TSA-seq scores for each gene, where higher TSA-seq (tyramide signal amplification sequencing) scores represent closer proximity to speckles,^23^ were compared with splicing factor signals at TSSs in K562 cells. We observed that the signals of SF3B1, U2AF1, and U2AF2 at TSSs of intron-containing and intronless genes did not correspond to the gene’s proximity to nuclear speckles (Figure S2A). In addition, we performed CUT&RUN on SRSF1, which localizes to nuclear speckles, and SRSF3, which does not. We found that both bind to TSSs, demonstrating that nuclear speckle localization is not required for TSS binding (Figures S2B and S2C).

Like TSSs, some enhancers are transcriptionally active, so we examined whether SF3B1, U2AF1, and U2AF2 bind to active enhancers. Their levels moderately correlated with RNAPIIS2P at enhancers (Figure S2D), suggesting that these splicing factors associate with active transcription sites independent of nearby 5′ splice sites.

Strikingly, the localization of U2AF2 is more complex. Expanding the view of chromatin profiling across the length of genes, we noted that U2AF2 is broadly distributed within the bodies of the most active genes (Figures 1A, top, and 1D), a pattern not observed in published chromatin immunoprecipitation sequencing (ChIP-seq) data (Figure 1E).^24^ U2AF2 CUT&RUN binding is as clear-cut as for any DNA-binding protein such as the transcription factors CTCF, MYC, and Max (Figure 1F). Notably, U2AF2 exhibits similar level of occupancy as CTCF, one of the most stably bound transcription factors. Despite intronless genes having similar expression levels, as demonstrated by comparable RNAPIIS2P occupancy on both gene types (Figure S3A), U2AF2 showed substantially higher occupancy on intron-containing gene bodies than on intronless gene bodies (Figure S3B). Therefore, in contrast to the promoter localization of splicing factors, U2AF2 association with gene bodies is specific to intron-containing genes (Figures 1A and 1D), suggesting U2AF2 occupancy depends on active splicing.

### U2AF2 binds to chromatin in intron-containing gene bodies

**Figure 2.**
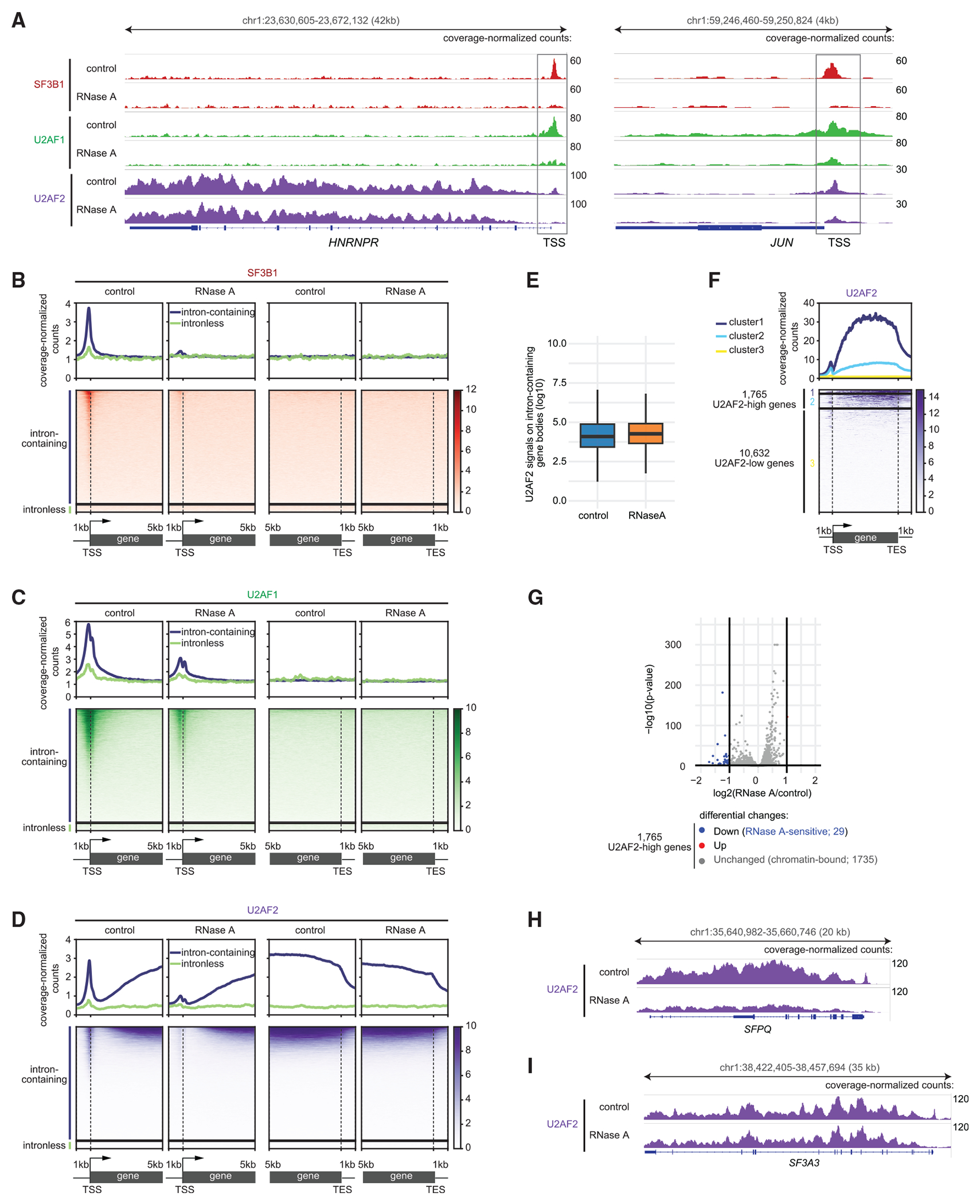
U2AF2 binds to chromatin in intron-containing gene bodies (A) Browser track showing the distribution of SF3B1, U2AF1, and U2AF2. (B–D) Heatmaps (bottom) and average plots (top) aligned to the TSS or TES of intron-containing and intronless genes, displaying coverage-normalized counts for SF3B1 (B), U2AF1 (C), and U2AF2 (D) under the indicated conditions. (E) U2AF2 coverage-normalized counts on intron-containing gene bodies under the indicated conditions. (F) U2AF2 coverage-normalized counts across intron-containing genes, grouped by k-means clustering (k = 3). (G) Volcano plot showing 1,765 U2AF2-high genes differentially enriched for U2AF2 after RNase A treatment compared with control (log_2_[RNase A/control] > 1, −log_10_[*p* value] < 0.05, *n* = 3). Genes with reduced U2AF2 (RNase A-sensitive) are highlighted in blue, chromatin-bound genes in gray, and one up-regulated gene in red. (H and I) Browser track showing U2AF2 on the RNase A-sensitive gene *SFPQ* (H) and the chromatin-bound gene *SF3A3* (I). See also Figure S3.

Active promoters contain nascent RNAs on the transcriptional machinery. We reasoned that promoter-localized splicing factors might directly bind to the promoter, or they might bind nascent RNAs and thereby bring antibody-tethered MNase close enough to DNA to produce CUT&RUN signals. To distinguish these possibilities, we enzymatically digested RNAs with RNase A prior to the antibody binding step targeting the splicing factor in CUT&RUN profiling. We observed strongly decreased signals of SF3B1, U2AF1, and U2AF2 at TSSs of genes upon RNA removal (Figures 2A–2D). However, RNAPIIS2P signals on chromatin were unaffected by RNase A treatment (Figure S3C), consistent with the stable binding of the RNAPII subunit RPB1.^25^ These results demonstrated that the association of these splicing factors with TSSs is RNA-mediated. Surprisingly, unlike at TSSs, no loss of U2AF2 signals on intron-containing gene bodies was observed following RNase A treatment, with median U2AF2 signals of 4.09 in the control and 4.27 in the RNase A condition (Figures 2A, left, 2D, and 2E). This shows that U2AF2 binds directly to chromatin in gene bodies. To determine whether U2AF2 binds to a subset of gene bodies through RNAs, we conducted k-means clustering analysis on 12,397 intron-containing genes. This analysis identified 1,765 genes with high U2AF2 signal (U2AF2-high genes) and 10,632 genes with low signal (U2AF2-low genes) (Figure 2F).

We then asked if any of the 1,765 U2AF2-high genes lose U2AF2 signal after RNase A treatment. Differential analysis revealed that RNase A treatment decreased U2AF2 signal on the bodies of only 29 (1.6%) genes (RNase A-sensitive genes), while no significant changes were detected on the other 1,735 U2AF2-high genes (chromatin-bound genes) (Figure 2G). A representative example of both an RNase A-sensitive gene and a chromatin-bound gene is shown in Figures 2H and 2I.

To further describe the localization of U2AF2 on chromatin in gene bodies, we mapped its distribution within a 10-kb region around the TSSs of the 1,735 chromatin-bound genes. We found that U2AF2 was mostly absent from the regions immediately downstream of TSSs (Figure S3D). Further investigation revealed that U2AF2 signals on chromatin begin to accumulate from the second exon of its targeted gene (Figures S3E and S3F). This localization suggests that direct U2AF2 binding to chromatin is linked to active splicing.

### RNAPII is not required to maintain U2AF2 on chromatin

We next investigated whether transcription is required to maintain U2AF2 on chromatin. To test this, we used transcription inhibitors to block transcription at different stage.^26^ Triptolide prevents transcription initiation by covalently binding to the XPB DNA helicase and inhibiting its ATPase activity required to translocate DNA into the active site of RNAPII.^27^ After initiation, several kinases phosphorylate RNAPII at serine-5, then RNAPII frequently pauses 0.3–3 kb downstream of TSSs *in vivo*.^28^ CDK9 kinase releases RNAPII into gene bodies for elongation by phosphorylating RNAPII at serine-2,^29,30^ but flavopiridol blocks this release by inhibiting CDK9 activity.^31^ While triptolide and flavopiridol inhibit transcription at specific stages, actinomycin D directly inhibits it by intercalating into DNA and acting as a roadblock to RNAPII progression.

**Figure 3.**
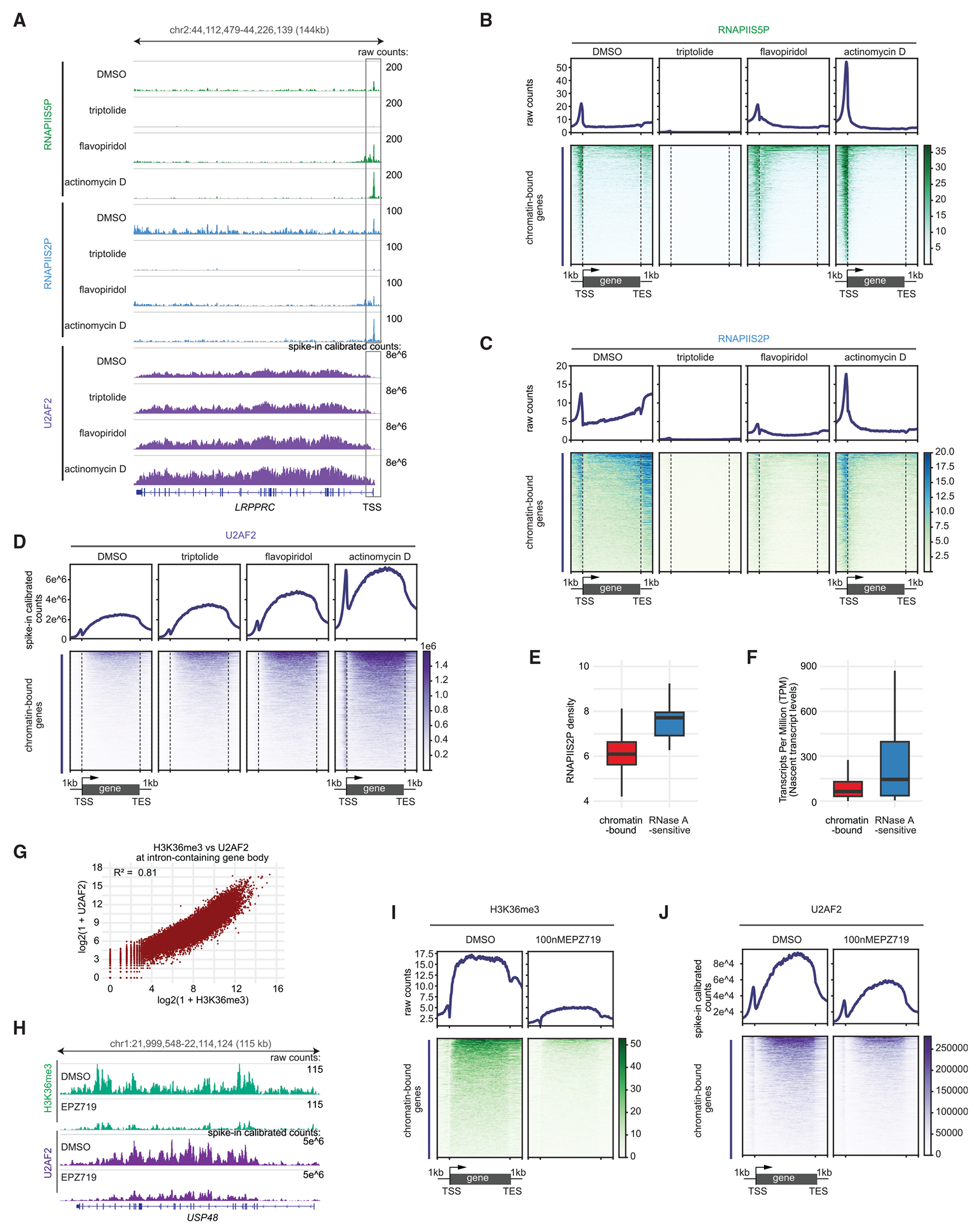
U2AF2 requires the H3K36me3 mark but not RNAPII to remain on chromatin (A) Browser track showing raw counts for RNAPIIS5P and RNAPIIS2P and spike-in calibrated counts for U2AF2. (B–D) Heatmaps (bottom) and average plots (top) showing raw counts for RNAPIIS5P (B), RNAPIIS2P (C), and spike-in calibrated counts for U2AF2 (D) on U2AF2 chromatin-bound genes under the indicated treatments. See also Figure S4. (E) RNAPIIS2P density on chromatin-bound or RNase A-sensitive genes. RNAPIIS2P RPK = total RNAPIIS2P mapped fragment number on regions including 1 kb upstream and downstream of the gene (gene length in kilobases). RNAPIIS2P density is log_2_(1 + RNAPIIS2P RPK). (F) Nascent transcript levels from chromatin-bound and RNase A-sensitive genes were quantified using published transcripts per million (TPM) values from chromatin fraction total RNA-seq data.^24^ (G) Scatterplots showing positive correlations between the log_2_-transformed total mapped fragment numbers of H3K36me3 and U2AF2 on gene bodies. Each dot represents a single gene. (H) Browser track showing raw counts for H3K36me3 and spike-in calibrated counts for U2AF2. (I and J) Heatmaps (bottom) and average plots (top) showing raw counts for H3K36me3 (I) and spike-in calibrated counts for U2AF2 (J) on U2AF2 chromatin-bound genes under the indicated treatments.

To confirm that these inhibitors block transcription after 1 h in K562 cells, we used CUT&Tag (cleavage under targets and tagmentation) to profile paused RNAPII phospho-serine-5 (RNAPIIS5P) and elongating RNAPIIS2P. CUT&Tag libraries for DMSO control and drug-treated samples were pooled at equal volumes to ensure comparable input. Raw read counts, representing the original input, were used to assess drug-induced RNAPII changes across the genome. Treatment with 10 μM triptolide resulted in strong reductions in both RNAPIIS5P (Figures 3A and 3B) and RNAPIIS2P (Figures 3A and 3C) on U2AF2 chromatin-bound genes, confirming impaired transcription initiation. Treatment with 1 μM flavopiridol did not affect RNAPIIS5P but decreased RNAPIIS2P levels on U2AF2 chromatin-bound gene bodies (Figures 3A–3C), confirming impaired transcription elongation. Treatment with 5 μg/mL actinomycin D increased RNAPIIS5P and RNAPIIS2P levels at TSSs but reduced RNAPIIS2P at chromatin-bound gene bodies (Figures 3A–3C), confirming halted RNAPII movement along the genes. RNAPIIS5P and RNAPIIS2P on RNase A-sensitive genes showed a similar response to these inhibitors as chromatin-bound genes (Figures S4A–S4C). These results confirm that these inhibitors block transcription as expected.

To determine if transcription inhibition affects U2AF2 occupancy genome-wide, we used CUT&RUN to profile U2AF2 and compared spike-in calibrated reads between DMSO and drug-treated samples. We found that, unlike RNAPII, U2AF2 levels slightly increased on chromatin-bound gene bodies upon triptolide treatment (Figures 3A and 3D), suggesting that once U2AF2 binds to chromatin, it does not require the persistent presence of RNAPII. Actinomycin D caused a dramatic increase in U2AF2 levels on both TSSs and gene bodies. This increase was not due to elevated levels at TSSs, as triptolide and flavopiridol had no effect there (Figure 3D). U2AF2 localizes to both nuclear speckles and nucleoplasm,^19^ and actinomycin D treatment increases its concentration in these speckles.^32^ This raised the possibility that genes closer to nuclear speckles, where U2AF2 is concentrated, would show higher U2AF2 gene body occupancy. However, we found no correspondence between U2AF2 occupancy on gene bodies and a gene’s proximity to nuclear speckles (Figure S4D), suggesting that U2AF2 binding to a gene body is independent of its distance from nuclear speckles. In addition, U2AF2 directly binds to the phosphorylated RNAPII C-terminal domain (CTD),^2^ but only ~34% of RNAPIIS2P signals co-localize with nuclear speckles,^33^ further evidence that U2AF2 binding occurs independently of nuclear speckle proximity.

To assess the impact of transcription inhibition on their genome-wide occupancy, we compared spike-in calibrated reads for SF3B1 and U2AF1 between DMSO and drug-treated samples. Actinomycin D treatment increased SF3B1 and U2AF1 signals at TSSs of both intron-containing and intronless genes, while triptolide and flavopiridol had no effect (Figures S4E and S4F). Previous studies have shown that actinomycin D increases RNAPII’s residence time on chromatin,^34^ which may enhance RNA-dependent splicing factor association with TSSs by prolonging the residence time of nascent RNAs bound to stalled RNAPII.

We propose that chromatin-bound U2AF2 transfers to newly synthesized pre-mRNA for splice-site recognition as RNAPII progresses, and when RNAPII stalls, U2AF2 returns to chromatin. Consistent with dynamic transfer from chromatin to nascent RNA, RNase-sensitive genes exhibited higher RNAPIIS2P occupancy (Figure 3E) and nascent transcript levels (Figure 3F) than chromatin-bound genes. Moreover, increased U2AF2 occupancy on RNase-sensitive gene bodies after RNA synthesis inhibition and enhanced RNAPII stalling by actinomycin D suggest that U2AF2 returns to chromatin when RNAPII stalls (Figure S4G). Therefore, persistent RNAPII transcription is not required to maintain U2AF2 on chromatin.

### H3K36me3 is required for U2AF2 chromatin binding

To address the basis for selectivity of chromatin binding by U2AF2, we performed CUT&Tag profiling on H3K36me3, which is known to bind preferentially to active intron-containing gene bodies.^35^ H3K36me3 is also enriched at exons compared with introns within the same genes.^7,36,37^ We observed a strong positive correlation between U2AF2 and H3K36me3 total abundance on intron-containing gene bodies (Figure 3G). Therefore, we reasoned that if U2AF2 binds to this histone mark, depleting it would reduce U2AF2 binding. To deplete H3K36me3, we treated cells with the selective SETD2 inhibitor EPZ-719,^38^ where SETD2 tri-methylates H3K36. After 24 h of 100 nM EPZ-719 treatment, H3K36me3 levels decreased on U2AF2 chromatin-bound genes (Figures 3H and 3I), leading to reduced U2AF2 occupancy (Figures 3H and 3J), while RNAPIIS2P levels remained unchanged (Figures S4H and S4I). These findings demonstrate that H3K36me3 is required to retain U2AF2 on chromatin without altering RNAPIIS2P occupancy.

As both U2AF2 and SETD2 bind to the phosphorylated CTD of RNAPII,^2,39^ U2AF2 may transfer from the CTD to gene bodies through the H3K36me3 mark deposited by SETD2 during transcription elongation. The H3K36me3 mark has a long half-life on chromatin^40^ and persists on chromatin after 1 h of triptolide treatment.^41^ Therefore, although RNAPIIS2P levels were reduced on gene bodies after 1 h of triptolide treatment, U2AF2 binding would still have been maintained through the H3K36me3 mark.

### U2AF2 preferentially binds to highly expressed exon-dense genes

**Figure 4.**
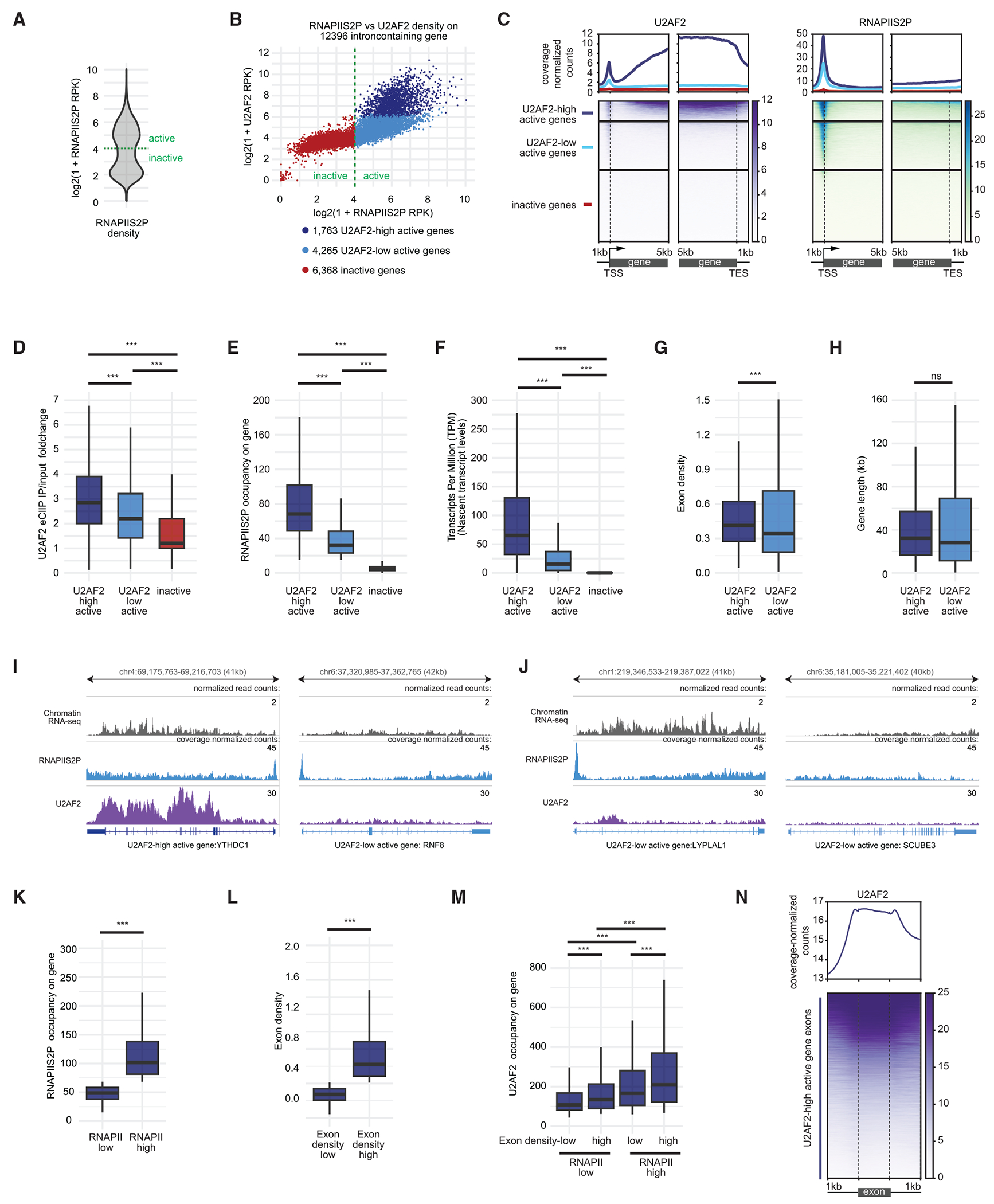
U2AF2 preferentially binds to exons of highly expressed exon-dense genes (A) Violin plot showing the distribution of RNAPIIS2P density across 12,396 intron-containing genes, calculated as log_2_(1 + RNAPIIS2P RPK). Genes were classified as active or inactive using an RNAPIIS2P density threshold of 4. (B) Scatterplots showing the relationship between RNAPIIS2P and U2AF2 density across intron-containing genes. Each dot represents a gene, with dark blue, light blue, and red dots indicating 1,763 U2AF2-high active, 4,265 U2AF2-low active, and 6,368 inactive genes, respectively. See also Figure S5. (C) Heatmaps (bottom) and average plots (top) aligned to U2AF2-high active, U2AF2-low active, and inactive genes, displaying coverage-normalized counts for U2AF2 and RNAPIIS2P. See also Figure S5. (D–H) Boxplots showing U2AF2 levels on transcripts (calculated as U2AF2 eCLIP IP/input foldchange) (D), RNAPIIS2P occupancy (RNAPIIS2P RPK) (E), nascent transcript levels (TPM) (F), exon density (G), and gene length (H) within each indicated gene group. U2AF2 eCLIP data were obtained from published sources.^42^ U2AF2 eCLIP IP/input foldchange = U2AF2 IP fragments/input fragments. Exon density = total exon number/gene length in kilobases. (I and J) Browser tracks showing coverage-normalized counts for U2AF2 and RNAPIIS2P and normalized signals for RNA-seq data. (K) Boxplots showing RNAPIIS2P occupancy on RNAPII-low and RNAPII-high genes, grouped by quartiles of RNAPIIS2P RPK. (L) Boxplots showing exon density values for exon density-low and exon density-high genes, grouped by quartiles of exon density. (M) Boxplots showing U2AF2 occupancy (U2AF2 RPK) on RNAPII-low and RNAPII-high genes with low or high exon density. Mann-Whitney-Wilcoxon test with Bonferroni correction was used. Adjusted *p* value: < 0.01 *, < 0.001 **, < 0.0001 ***. (N) Heatmaps (bottom) and average plots (top) showing U2AF2 coverage-normalized counts on exons of U2AF2-high active genes, extending 1 kb upstream and downstream of exons.

To investigate whether U2AF2 preferentially accumulates on chromatin in specific actively expressed genes, we categorized these genes by their RNAPIIS2P density along their length and examined U2AF2 density and distribution across them. We classified 6,028 genes as active and 6,368 as inactive (Figure 4A). Among them, 1,763 exhibited high U2AF2 levels (U2AF2-high active genes) and 4,265 had low U2AF2 levels (U2AF2-low active genes), while genes with undetectable RNAPIIS2P had low U2AF2 levels (inactive genes) (Figures 4B and 4C).

To investigate whether higher U2AF2 occupancy on chromatin leads to higher U2AF2 levels on transcripts, we analyzed published enhanced cross-linking immunoprecipitation (eCLIP) data to measure U2AF2 levels on transcripts.^42^ Consistent with the pattern observed for U2AF2 occupancy on chromatin, median U2AF2 levels were highest on U2AF2-high active gene transcripts, lower on U2AF2-low active gene transcripts, and lowest on inactive gene transcripts (Figure 4D). This consistent pattern suggests a bidirectional relationship: U2AF2 binding to transcripts may enhance its association with chromatin, or vice versa. However, since U2AF2 binds to chromatin largely independent of RNA, the increased U2AF2 levels on chromatin are not due to its binding to chromatin-associated transcripts. Instead, U2AF2 binding to chromatin would drive greater U2AF2 binding to these transcripts, promoting co-transcriptional splicing.

To examine why U2AF2 preferentially binds to a subset of active genes, we compared the expression and structures of U2AF2-high active and U2AF2-low active genes. U2AF2-high active genes showed higher RNAPIIS2P occupancy (Figure 4E) and greater nascent transcript levels (Figure 4F), based on Encyclopedia of DNA Elements (ENCODE) RNA sequencing (RNA-seq) data from the chromatin fraction.^24^ They also had higher exon density despite having similar gene lengths to U2AF2-low active genes (Figures 4G and 4H). For example, the U2AF2-high active gene *YTHDC1* exhibited higher RNAPIIS2P occupancy, transcript levels, and exon density compared with the U2AF2-low active gene *RNF8* (Figure 4I).

We wondered if both high transcription and high exon density are required for U2AF2 chromatin binding. The *LYPLAL1* gene, which had high RNAPIIS2P occupancy and transcript levels similar to the *YTHDC1* gene but low exon density, showed low U2AF2 occupancy (Figure 4J). Likewise, *SCUBE3*, with comparable exon density but lower RNAPIIS2P occupancy and transcript levels than *YTHDC1*, also had low U2AF2 occupancy (Figure 4J). Based on this, we hypothesized that U2AF2 occupancy would be higher on U2AF2-high active genes with both higher RNAPIIS2P occupancy and exon density. To test this, we divided U2AF2-high active genes into two groups based on RNAPIIS2P occupancy quantiles (Figure 4K) or exon density quantiles (Figure 4L). U2AF2 occupancy increased with higher exon density, regardless of RNAPIIS2P levels, and with higher RNAPIIS2P occupancy, regardless of exon density. Notably, genes with both high RNAPIIS2P occupancy and high exon density had the highest U2AF2 occupancy (Figure 4M). These findings suggest that U2AF2 preferentially binds to highly transcribed, exon-dense genes.

### U2AF2 preferentially binds to exons

Since U2AF2 preferentially binds to exon-dense active genes, we expected that it would be enriched on exons, as observed (Figure 4N). Indeed, we found that U2AF2 occupancy was higher on exons than introns in both U2AF2-high and U2AF2-low active genes. Additionally, U2AF2 levels steadily increased from the 5′ to the 3′ ends on successive exons and introns in U2AF2-high active genes (Figure S5A).

Next, we investigated what features of exon DNA might contribute to U2AF2’s enrichment on exons. Exon DNA has a higher GC content and nucleosome occupancy than intron DNA.^7,12,13^ To determine if this pattern holds for U2AF2-high active and U2AF2-low active genes, we measured the GC content and nucleosome occupancy of their first 10 exons and introns. Exons in both groups had higher GC content than introns, but exons in U2AF2-high active genes had lower GC content compared with exons in U2AF2-low active genes (Figure S5B). Using published MNase-seq data,^43^ we observed higher nucleosome occupancy in exons than in introns, based on nucleosome-sized fragment (≥150 bp) densities in both gene groups (Figure S5C). To assess whether the reduced nucleosome occupancy in introns is due to lower GC content, we normalized nucleosome occupancy by calculating nucleosome-sized fragments per 1,000 GC base pairs (nucleosome occupancy/kGC). Even after normalization, exons still showed higher nucleosome occupancy than introns (Figure S4D), indicating factors beyond GC content contribute to increased nucleosome occupancy in exons. However, once normalized for GC content, the difference in nucleosome occupancy between U2AF2-high active and U2AF2-low active exons disappeared (Figure S5D), suggesting that the lower nucleosome occupancy in U2AF2-high active genes is primarily due to their reduced GC content.

As the predominant mode of splicing in vertebrates is exon definition, where the spliceosome first recognizes and assembles across an exon,^44,45^ elevated U2AF2 on exon DNA suggests that it may aid in exon recognition.

### Chromatin-bound U2AF2 enhances exon inclusion in highly expressed genes

To determine if U2AF2 is required for splicing fidelity in active genes, we analyzed splicing errors upon U2AF2 depletion using published RNA-seq data from U2AF2 knockdown (U2AF2 KD) and wild-type (WT) K562 cells.^42^ Of 6,028 active genes, 3,942 had available percent-splice-in (PSI) values—calculated as the ratio of inclusion read density to the sum of inclusion and exclusion read densities—and *p* values for their exons, introns, or splice sites, which are required for identifying splicing errors after U2AF2 KD.

**Figure 5.**
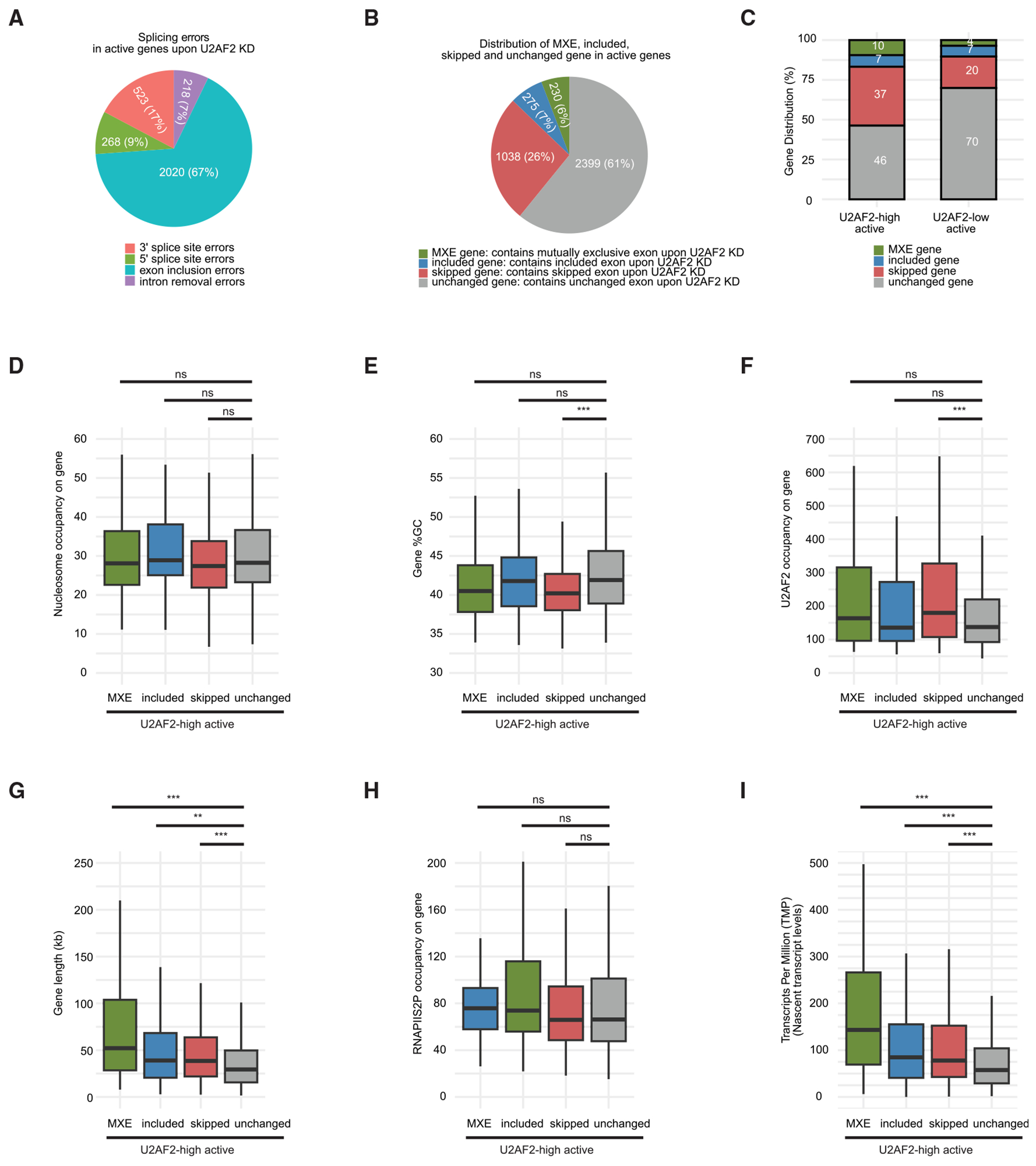
Chromatin-bound U2AF2 enhances exon inclusion in highly expressed genes (A) Pie chart showing the proportion of splicing errors in active genes after U2AF2 KD. (B) Pie chart showing the distribution of unchanged, skipped, included, and MXE genes in active genes. (C) Percentage of unchanged, skipped, included, and MXE genes in the U2AF2-high active and U2AF2-low active gene group. (D–I) Boxplots showing nucleosome occupancy (D), % GC (E), U2AF2 occupancy (U2AF2 RPK) (F), gene length (G), RNAPIIS2P occupancy (RNAPIIS2P RPK) (H), and nascent transcript levels (TPM) (I) in MXE, included, skipped, and unchanged genes in the U2AF2-high active gene group. Nucleosome occupancy = total number of nucleosomal fragments (≥150 bp) on regions including 1 kb upstream and downstream of the gene (gene length in kilobases). % GC = 100 * total number of GC bases/gene length in base pairs. Adjusted *p* value: < 0.01 *, < 0.001 **, < 0.0001 ***. See also Figure S6.

We identified 3,029 significant splicing errors upon U2AF2 KD, where the PSI values for exons, introns, or splice sites in WT and U2AF2 KD cells showed significant differences, with *p* values below 0.05. Notably, 67% of these errors were exon inclusion errors (Figure 5A), suggesting that U2AF2 is primarily required for correct exon inclusion. We then investigated how many active genes require U2AF2 for correct exon inclusion. If an exon’s PSI value is significantly higher in WT than in U2AF2 KD cells, it means the exon is skipped in U2AF2 KD cells, and if the PSI is significantly lower in WT, the exon is included. Upon U2AF2 KD, 61% of the 3,942 active genes showed no changes in exon inclusion (unchanged genes), 26% had skipped exons (skipped genes), 7% had included exons (included genes), and 6% exhibited mutually exclusive exons (MXE genes) (Figure 5B). Overall, U2AF2 is required for correct exon inclusion in 39% of the 3,942 active genes. We examined whether higher gene expression improves the detection of exon inclusion errors due to increased RNA-seq reads providing better *p* values. However, we found no correlation between gene expression (calculated as [log2(1 + FPKM)], where FPKM is fragments per kilobase of transcript per million mapped reads) and *p* values for skipped, included, or unchanged exons, indicating that the detection of exon inclusion errors was not biased toward higher-expressed genes (Figure S6A).

To obtain independent validation for U2AF2 function, we assessed whether U2AF2 is essential for exon inclusion fidelity in HepG2 cells for the same set of genes active in K562 cells, using ENCODE RNA-seq data from U2AF2 KD and WT HepG2 cells.^24^ In HepG2, of the 3,916 active genes with available PSI and *p* values for their exons, 57%, 28%, 9%, and 7% were classified as unchanged, skipped, included, and MXE genes, respectively. This shows that U2AF2 is required for correct exon inclusion in 43% of active genes (Figure S6B). Additionally, 65% (940 of 1,440) of genes with exon inclusion errors in K562 also had errors in HepG2, while 66% (1,307 of 1,985) of genes without errors in K562 showed no errors in HepG2 after U2AF2 KD (Figure S6C). These results confirm the consistent role of U2AF2 in maintaining exon inclusion accuracy across different cell lines.

To determine if U2AF2 chromatin binding promotes exon inclusion fidelity, we analyzed the percentage of unchanged, skipped, included, and MXE genes in U2AF2-high active and U2AF2-low active genes. If chromatin-bound U2AF2 is essential, we would expect more exon inclusion errors in U2AF2-high active genes after U2AF2 KD. Indeed, in K562, 54% of U2AF2-high active genes (811 of 1,510) showed exon inclusion errors, compared with 30% of U2AF2-low active genes (732 of 2,432) (Figure 5C). Similarly, in HepG2, 52% of U2AF2-high active genes (743 of 1,435) had exon inclusion errors, compared with 39% of U2AF2-low active genes (959 of 2,481) after U2AF2 KD (Figure S6D). These results suggest that chromatin-bound U2AF2 enhances exon inclusion fidelity. Additionally, 37% and 36% of U2AF2-high active genes showed exon skipping in K562 and HepG2, respectively, after U2AF2 KD, demonstrating that chromatin-bound U2AF2 primarily enhances exon inclusion in its target genes (Figure 5C; Figure S6D).

To test if expression-matched U2AF2-high active genes had a higher percentage of skipped genes compared with U2AF2-low active genes, we divided the 3,942 active genes in K562 into 10 groups based on RNA-seq FPKM deciles. U2AF2-high and U2AF2-low active genes showed similar expression levels at each decile, except for deciles 8 and 10 (Figure S6E). U2AF2 total levels increased gradually with RNAPIIS2P levels (Figure S6F), suggesting a continuum of U2AF2 gene-body binding. Consequently, U2AF2 occupancy on both U2AF2-high active and U2AF2-low active gene classes increased with higher expression levels (Figure S6G). As U2AF2 occupancy increased, the percentage of skipped genes in both U2AF2-high active and U2AF2-low active gene classes also increased upon U2AF2 KD (Figure S6H). Additionally, U2AF2-high active genes had a higher percentage of skipped genes compared with U2AF2-low active genes, despite similar expression levels at deciles 2, 3, 5, 6, 7, and 9. For example, at decile 6, 41% of U2AF2-high active genes had skipped exons, compared with 30% of U2AF2-low active genes upon U2AF2 KD (Figure S6H). These results confirm and extend our conclusion that chromatin-bound U2AF2 is required for exon inclusion.

We examined nucleosome occupancy in skipped, included, and MXE genes within the U2AF2-high active gene group. All groups had similar nucleosome occupancy, implying that nucleosome occupancy does not influence exon skipping upon U2AF2 KD (Figure 5D). However, skipped genes had lower GC content (Figure 5E) and higher U2AF2 occupancy (Figure 5F) compared with unchanged genes.

Next, we explored the features contributing to the exon inclusion errors in skipped, included, and MXE genes within the U2AF2-high active gene group upon U2AF2 KD. These genes had median lengths ranging from 40 to 50 kb, at least 1.3 times longer than unchanged genes, which had a median length of approximately 30 kb (Figure 5G). Although RNAPIIS2P occupancy was similar across these gene groups (Figure 5H), the median levels of nascent transcripts in skipped, included, and MXE genes were ~1.5 times higher than in unchanged genes (Figure 5I). Taken together, these findings suggest that U2AF2’s preferential binding to highly expressed genes enhances exon inclusion in these genes.

### Chromatin-bound U2AF2 enhances exon selection accuracy in splicing factor genes

**Figure 6.**
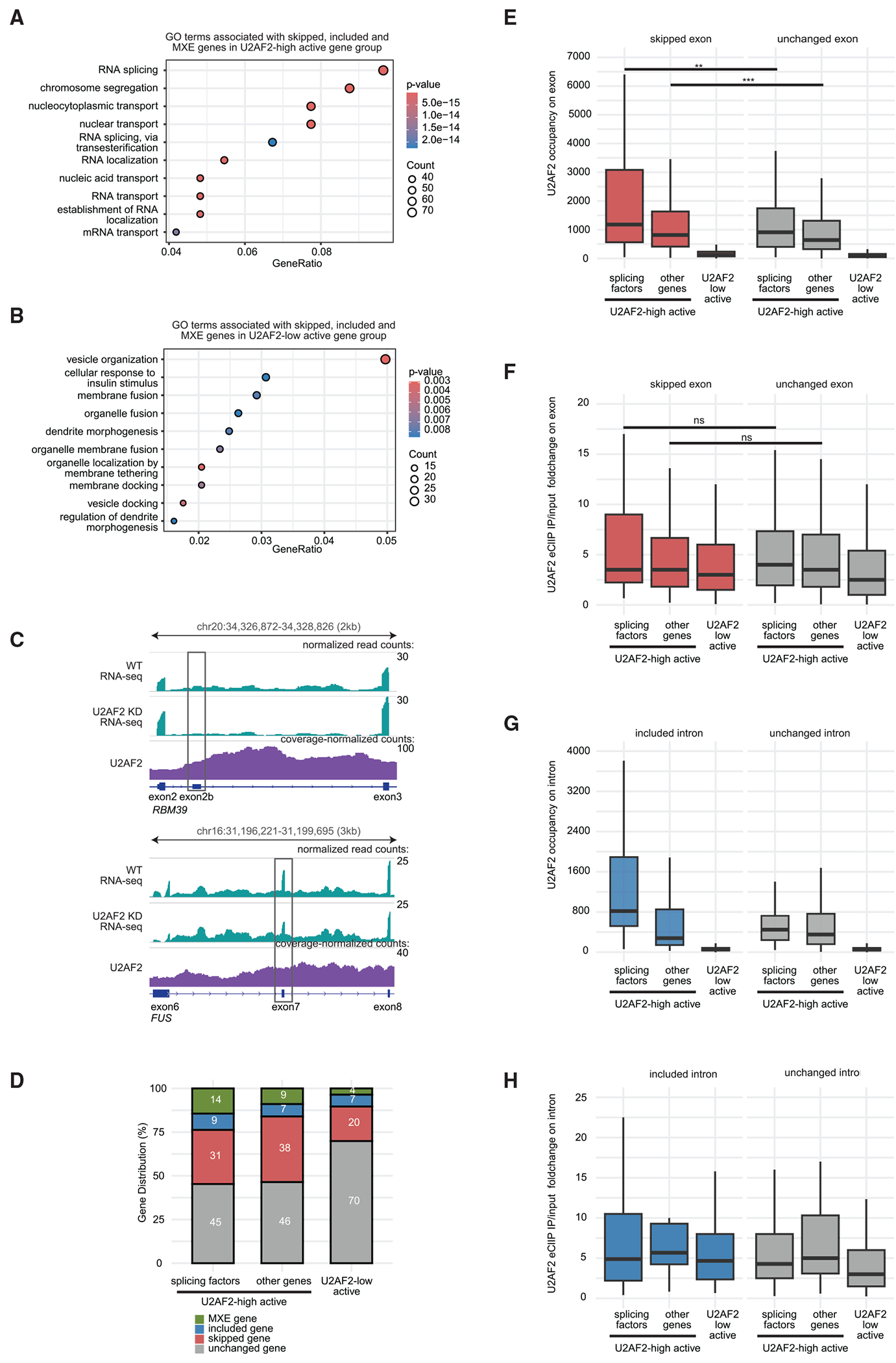
Chromatin-bound U2AF2 enhances exon selection accuracy in splicing factor genes (A and B) Dot plots showing the top 10 GO terms for U2AF2-high active (A) and U2AF2-low active genes (B) with skipped and/or included exons upon U2AF2 KD. Dot size indicates the number of associated genes, and color represents the *p* value adjusted using the Benjamini-Hochberg method. (C) Distribution of transcript signal in WT and U2AF2 KD^42^ and U2AF2 CUT&RUN signal on *RBM39* and *FUS* gene in WT. (D) Percentage of unchanged, skipped, included, and MXE genes in U2AF2-high and U2AF2-low active gene groups. (E and F) Comparison of U2AF2 levels on exonic DNA (U2AF2 RPK) (E) and RNA (U2AF2 eCLIP IP/input fold change) (F). (G and H) Comparison of U2AF2 levels on intronic DNA (U2AF2 RPK) (G) or RNA (U2AF2 eCLIP IP/input foldchange) (H). See also Figure S7.

To investigate whether U2AF2-high active and U2AF2-low active genes with exon inclusion errors upon U2AF2 KD are associated with specific biological processes, we performed Gene Ontology (GO) analysis on the combined list of skipped, included, and MXE genes in these two gene groups. Strikingly, 9% (76 of 811) of U2AF2-high active genes with exon inclusion errors were linked to the RNA splicing category (Figure 6A). Further inspection showed that U2AF2 binds to the chromatin in genes encoding HNRNPs (e.g., *HNRNPC, HNRNPR*, and *HNRNPLL*) and key spliceosome proteins (e.g., *SNRNP70, SF3B1, SF3A3, U2AF1, SF1*, and *AQR*), regulating correct exon inclusion in these splicing factor genes. By contrast, no enrichment of splicing-relevant GO categories was observed in U2AF2-low active genes with exon inclusion errors (Figure 6B). Splicing factors can autoregulate their expression through negative feedback loops by binding to their own pre-mRNA and controlling their splicing.^46–48^ For example, the RBM39 splicing factor autoregulates by including a poison exon in its mRNA, producing a nonproductive isoform.^49^ Similarly, the splicing factor FUS binds to its own poison exon, promotes its inclusion, and produces a nonproductive transcript.^50^ Notably, we found that U2AF2 was enriched at the poison exons of both the *RBM39* and *FUS* genes, and the inclusion levels of these exons are reduced upon U2AF2 KD (Figure 6C).

We next investigated whether U2AF2 regulates the splicing of splicing factor transcripts by preferentially binding to chromatin in these genes. Since 139 of the 1,510 U2AF2-high active genes are involved in RNA splicing, we divided the U2AF2-high active genes into 139 splicing factor genes and 1,371 other genes. Using the available PSI values for exons, we found that 55% (76 of 139) of splicing factor genes had exon inclusion errors, with 31% showing skipped exons upon U2AF2 KD (Figure 6D). Additionally, using the PSI values for introns, we found that 29% (15 of 51) of splicing factor genes had intron removal errors, with 24% retaining introns upon U2AF2 KD (Figure S7A). Splicing factor genes exhibited the highest U2AF2 chromatin occupancy (Figure S7B) and the highest levels of new pre-mRNA transcripts (Figure S7C) compared with other genes and U2AF2-low active genes. The regulation of RNA splicing also involves *cis*-acting RNA sequences, including 5′ and 3′ splice sites.^51,52^ However, the similar 5′ and 3′ splice site strength scores among splicing factor genes, other genes, and U2AF2-low active genes (Figures S7D and S7E) suggest that these strengths do not explain the differences in splicing error frequency observed in these gene groups upon U2AF2 KD. Therefore, we conclude that U2AF2’s preferential binding enhances exon inclusion and intron removal in splicing factor genes. U2AF2 binding to chromatin positions it near newly synthesized transcripts at the transcription site, leading us to expect higher co-transcriptional splicing efficiency in transcripts from U2AF2-targeted genes. Indeed, we found that splicing factor gene transcripts exhibited higher co-transcriptional splicing efficiencies compared with U2AF2-low active gene transcripts (Figure S7F). Overall, our results suggest a network whereby chromatin-bound U2AF2 enhances splicing accuracy and efficiency in splicing factor gene transcripts.

Finally, we aimed to understand why certain exons and introns in splicing factor genes require U2AF2 for their inclusion and removal. We analyzed U2AF2 levels on both chromatin and transcripts to determine which binding events promote exon inclusion and intron removal in splicing factor genes. U2AF2 levels were higher on chromatin (Figure 6E) but not on transcripts (Figure 6F) at skipped exons in splicing factor genes compared with U2AF2-low active genes. Notably, in splicing factor genes, chromatin-bound U2AF2 was also higher at skipped exons than unchanged exons (Figure 6E), while transcript-bound U2AF2 levels remained similar (Figure 6F). This pattern was consistent in other genes, suggesting that U2AF2’s binding to exon DNA promotes exon inclusion. Similarly, included introns in splicing factor genes showed higher chromatin-bound U2AF2 (Figure 6G) but not transcript-bound U2AF2 (Figure 6H) compared with U2AF2-low active genes. Chromatin-bound U2AF2 was also more abundant on included introns than on unchanged introns in splicing factor genes (Figure 6G), suggesting that U2AF2 enrichment on intron DNA promotes intron removal in these transcripts. Overall, chromatin-bound U2AF2 enhances the inclusion of specific exons and removal of certain introns in a network of splicing factor transcripts, contributing to the balanced expression of splicing machinery. Thus, U2AF2 acts as a homeostatic regulator for maintaining splicing equilibrium and ensuring accurate exon inclusion.

## DISCUSSION

In this study, we demonstrate that the splicing factors SF3B1, U2AF1, and U2AF2 co-localize at active gene promoters via nascent RNAs, suggesting that they co-assemble with the transcriptional machinery during transcription initiation. Strikingly, U2AF2 distribution is extended to intron-containing gene bodies, directly binding to chromatin in an RNA-independent manner, and its maintenance on chromatin requires the H3K36me3 modification mark but not the persistent presence of RNAPII. Chromatin-bound U2AF2 is preferentially enriched at exons of highly expressed, exon-dense genes, enhancing exon inclusion accuracy.

**Figure 7.**
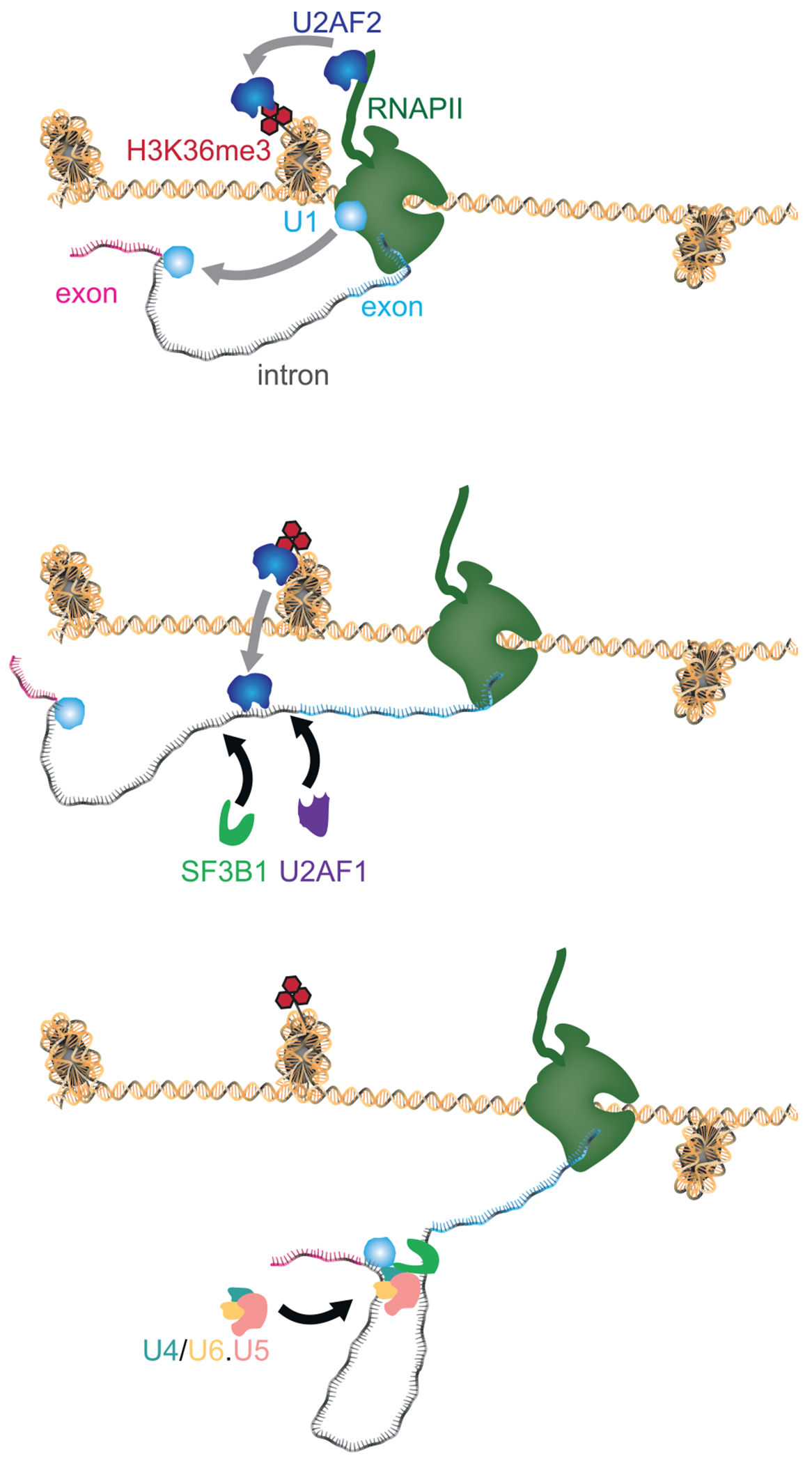
Chromatin-bound U2AF2 ensures exon inclusion (Top) RNAPII interacts directly with U1 snRNP, enabling the rapid recognition of the 5′ splice site in nascent pre-mRNA by U1 snRNP.^53^ U2AF2 initially binds to the RNAPII CTD^2,54^ and transfers from elongating RNAPII to chromatin through the H3K36me3 mark deposited by SETD2 during transcription elongation. (Middle panel) U2AF2 detaches from chromatin and transfers to the polypyrimidine tract on nascent pre-mRNA, stabilizing U2AF1 at the 3′ splice site and SF3B1 at the branchpoint to form the spliceosomal A complex.^1^ (Bottom) Recruitment of the preassembled U4/U6.U5 tri-snRNP complex forms the pre-catalytic B complex, the first fully assembled spliceosome with all five snRNPs.^55,56^ The B complex then undergoes extensive rearrangements to activate the spliceosome for splicing.^57,58^ Overall, U2AF2 binding to chromatin guides the spliceosome in recognizing splice junctions, ensuring efficient exon inclusion.

We propose a chromatin guide model, where U2AF2 bound to chromatin guides the spliceosome for quick and accurate co-transcriptional splicing during every round of transcription (Figure 7). As both U2AF2 and SETD2 bind to the RNAPII CTD,^2,39^ U2AF2 transfers from the CTD to chromatin through the H3K36me3 mark deposited by SETD2 during transcription elongation (Figure 7, top panel). By binding directly to chromatin, U2AF2 is positioned near nascent pre-mRNA transcripts. This proximity enables U2AF2 to transfer from chromatin to pre-mRNAs once they are synthesized by RNAPII, guiding the spliceosome to recognize splice sites in real time (Figure 7, middle and bottom panels). Therefore, chromatin-bound U2AF2 not only speeds up co-transcriptional splicing but also improves its accuracy. Notably, U2AF2 occupancy on chromatin increases, even in RNase A-sensitive genes where U2AF2-chromatin binding is RNA-dependent, when RNAPII is stalled and RNA synthesis is inhibited by actinomycin D. This suggests that after transferring to pre-mRNA, U2AF2 returns to chromatin as RNAPII stalls. This cycling keeps U2AF2 positioned near newly transcribed pre-mRNA, guiding the spliceosome during each round of transcription and ensuring the accuracy and efficiency of co-transcriptional splicing.

Approximately 95% of human multiexon genes undergo alternative splicing,^59,60^ but how the spliceosome selectively includes certain exons is not fully understood. Unlike genes in organisms with relatively compact genomes, which often have short introns spliced through intron definition, mammalian genes typically contain long introns that are predominantly spliced through exon definition.^5,61^ In the *in vitro* exon definition model, the spliceosome first assembles across an exon on the transcript, with U1 snRNP recognizing the downstream 5′ splice site, and U2AF2 and U2AF1 binding to the upstream polypyrimidine tract and 3′ splice site, respectively.^1,62,63^ However, given the complexity of human gene structures, low splice site sequence conservation,^64^ and our observation that U2AF2 binding to transcripts does not predict exon skipping upon U2AF2 KD, the exon definition model based solely on spliceosome binding to the transcript does not fully explain exon selection *in vivo*. Since exon selection occurs mostly co-transcriptionally, the spliceosome must accurately and quickly assemble on exons in nascent transcripts during every round of transcription. Therefore, an *in vivo* model is needed to elucidate how specific exons are accurately selected for inclusion co-transcriptionally. Indeed, the preferential binding of U2AF2 to both exonic DNA and the polypyrimidine tract near exonic RNA will guide the spliceosome to quickly recognize and select newly synthesized exonic RNA positioned close to its corresponding DNA. The persistent presence of U2AF2 ensures that the spliceosome consistently includes the correct exon in transcripts during each round of transcription. However, after U2AF2 KD, exons lack this guide on their DNA, reducing the spliceosome’s efficiency in recognizing them, and leading to exon skipping. Thus, chromatin-bound U2AF2 can guide splicing factors to accurately select exons for inclusion during transcription.

Chromatin-bound U2AF2 would also promote efficient co-transcriptional intron removal. The pre-mRNA intron is defined by the 5′ and 3′ splice sites at its ends. The direct interaction between U1 snRNP and RNAPII enables the U1 snRNP/5′ splice site complex to move with RNAPII, forming a pre-mRNA intron loop. This loop has been proposed to facilitate scanning of U2AF2 for the 3′ splice site^53,65^ thereby promoting efficient intron removal.^6,66^ However, instead of scanning for the 3′ splice site in pre-mRNA, U2AF2 binds to intronic DNA, positioning itself near the intronic RNA. This proximity allows U2AF2 to transfer from DNA to the polypyrimidine tract near the 3′ splice site, guiding the spliceosome to quickly and correctly pair the 3′ and 5′ splice sites for intron removal. After U2AF2 KD, this guide is absent, leading to intron retention. This is supported by our observations that U2AF2 chromatin occupancy enhances intron removal efficiency and accuracy.

Chromatin-bound U2AF2 would also regulate co-transcriptional splicing homeostasis. Tight regulation of the splicing factor network is crucial for maintaining splicing fidelity. To achieve this, splicing factor expression levels are commonly fine-tuned through a negative feedback loop.^46–49,67^ In this process, a splicing factor autoregulates the alternative splicing of its own transcripts or cross-regulates other splicing factor transcripts. While negative autoregulation has been linked to RNA sequence elements and alternative binding of splicing factors, how splicing factor network homeostasis is regulated co-transcriptionally remains unclear. Our study shows that U2AF2 preferentially binds chromatin at highly transcribed splicing factor genes, enhancing splicing accuracy and efficiency. Chromatin-bound U2AF2 finetunes this network in real time, ensuring the correct production of transcripts while they are still being transcribed. High U2AF2 levels create a feedback loop where correctly spliced splicing factor transcripts support ongoing spliceosome function, promoting splicing homeostasis. Thus, U2AF2 initiates a homeostatic mechanism that ensures proper spliceosome function and accurate exon inclusion.

### Limitations of the study

Detection of any factor on chromatin by CUT&RUN profiling depends on antibody specificity, yield, and epitope abundance, so that lack of detectable U2AF2 over most active gene bodies may be attributable to these limitations. U2AF2 total abundance increases gradually with increasing RNAPII (Figure S6F), raising the possibility that there is a continuum of U2AF2 gene-body binding and that our division into high and low classes to simplify the analysis is arbitrary. This would not affect our conclusions, and we expect that future work with more sensitive technologies will reveal the small fraction of genes and exons in which U2AF2 detectably binds and mediates exon inclusion to have been an underestimate. Since chromatin-bound U2AF2 regulates the splicing of other splicing factor transcripts, we cannot exclude the possibility that some exon inclusion and intron removal errors observed upon U2AF2 KD are due to indirect effects from misregulated binding or expression levels of other splicing factors. However, this would not alter our conclusions about chromatin-bound U2AF2, as U2AF2 KD results in a higher frequency of splicing errors in U2AF2-high active genes compared with U2AF2-low active genes, where lower levels of chromatin-bound U2AF2 are detected.

## RESOURCE AVAILABILITY

### Lead contact

Further information and requests for reagents should be directed to and will be fulfilled by the lead contact, Steven Henikoff (steveh@fredhutch.org).

### Materials availability

All materials generated in this study are available from the lead contact with a completed materials transfer agreement.

## STAR★METHODS

### EXPERIMENTAL MODEL AND STUDY PARTICIPANT DETAILS

#### Human cell culture

Human K562 cells (ATCC, Manassas, VA, Catalog #CCL-243) were cultured following the supplier’s protocol. For inhibitor treatments, the inhibitors’ stock solutions in DMSO were diluted in the primary medium and added directly to the cultures at the specified final concentrations. The transcription inhibitors used were Triptolide at 10 μM (Selleckchem, Catalog #S3604), Flavopiridol hydrochloride hydrate at 1 μM (Sigma-Aldrich, Catalog #FL3055), Actinomycin D at 5 μg/ml (Sigma-Aldrich, Catalog #A9415). DMSO at 1:1,000 v/v was used as control. Cells were harvested after 24 hours of 100nM EPZ-719 (MedChem Express, Catalog #HY-139626) treatment, or after 1 hour of treatment with Triptolide, Flavopiridol, or Actinomycin D and processed for CUT&RUN or CUT&Tag profiling.

### METHOD DETAILS

#### Antibodies

We used the following antibodies: Guinea Pig anti-Rabbit IgG (Heavy & Light Chain) (Antibodies Online, Cat# ABIN101961), H3K36me3 (Rabbit monoclonal anti-H3K36me3, Epicypher, Cat# 13-0058), RNAPIIS2P (Rabbit monoclonal anti-RNAPIIS2P, Cell Signaling Technology, Cat# 13499S), RNAPIIS5P (Rabbit monoclonal anti-RNAPIIS5P, Cell Signaling Technology, Cat# 13523S), SF3B1 (Rabbit monoclonal anti-SF3B1, Cell Signaling Technology, Cat# 14434S), U2AF1 (Rabbit polyclonal anti-U2AF35, Bethyl Laboratories, Cat# A302-079A) and U2AF2 (Rabbit polyclonal anti-U2AF65, Abcam, Cat# ab37530), SRSF1 (Rabbit polyclonal anti-SRSF1, Thermo Fisher Scientific, Cat# A302-052A) and SRSF3 (Rabbit polyclonal anti-SRSF3, MBL International, Cat#RN080PW). The final concentrations of the antibodies used in CUT&Tag or CUT&RUN are specified in the following sections.

#### CUT&Tag-direct for whole cells

CUT&Tag reactions were performed according to the CUT&Tag-direct protocol^75,76^ with some modifications. Briefly, 50,000 cells were spun down and resuspended in wash buffer (20 mM HEPES pH 7.5, 150 mM NaCl, 0.5 mM spermidine, 0.05% Triton-X100, Roche Complete Protease Inhibitor EDTA-Free tablet) by gentle pipetting. 5 μl of Bio-Mag Plus Concanavalin A (ConA)-coated magnetic beads (Bangs Laboratories Catalog #BP531) per sample were activated and added to the cells, then incubated for 10 min at room temperature. ConA-bound cells were suspended in antibody binding buffer (wash buffer containing 2 mM EDTA) and split into individual 0.5 ml tubes for overnight incubation with the primary antibody at a 1:10 dilution at 4°C. After removing unbound primary antibody by washing, the samples were resuspended in wash buffer containing the secondary antibody (guinea pig anti-rabbit IgG) at a 1:15 dilution and incubated at 4°C for 1 h. Following another wash, the samples were resuspended in 300-wash buffer (wash buffer with an additional 150 mM NaCl) containing Protein AG-Tn5 (pAG-Tn5 at a 1:10 dilution, EpiCypher, Catalog #15-1117) and incubated at 4°C for 1 h. The samples were then washed in 300-wash buffer and resuspended in tagmentation buffer (300-wash buffer with 10 mM MgCl_2_), followed by incubation at37°C for 1 h to complete the Tn5 tagmentation reaction. The samples were washed with TAPS wash buffer (10 mM TAPS with 0.2 mM EDTA) and resuspended in 5 μl of release solution (10 mM TAPS, 0.1% SDS, and 1:10 Thermolabile Proteinase K (New England Biolabs, Catalog #P8111S)). They were then incubated in a thermocycler with a heated lid at 37°C for 1 h and 58°C for 1 h to release fragments. To quench SDS, 4 μl of 1.5% Triton-X100 was added. PCR was performed by adding 2 μl each of barcoded 10 mM i5 and i7 primer solutions and 42 μl of premixed KAPA PCR Master Mix (10 μl of HiFi buffer, 1.5 μl of 10 mM dNTPs, 1 μl of KAPA HiFi polymerase, and 29.5 μl of H_2_O) (Roche, Catalog #07958846001). The following cycling conditions were used: Cycle 1: 58 °C for 5 min; Cycle 2: 72 °C for 5 min; Cycle 3: 98 °C for 30 s; Cycle 4: 98 °C for 10 s; Cycle 5: 60 °C for 10 s; Repeat Cycles 4–5 13 times; 72 °C for 1 min; Hold at 12 °C. CUT&Tag libraries were cleaned with HighPrep Paramagnetic bead-based post PCR clean-up reagent (MagBio, Catalog # AC-60500) at a 1.3:1 (vol/vol) ratio of beads to sample, quantified on a Agilent 4200 D1000 TapeStation (Agilent, Catalog #5067-5584) and pooled for sequencing.

#### CUT&RUN

CUT&RUN was carried out as previously described^16^ with some modifications. Briefly, 600,000 cells were spun down and resuspended in wash buffer (20 mM HEPES pH 7.5, 150 mM NaCl, 0.5 mM spermidine, Roche Complete Protease Inhibitor EDTA-Free tablet) and bound to activated Concanavalin A-coated magnetic beads for 10 min at room temperature. The ConA-bound cells were then split into PCR tubes, the supernatant was removed the cells were suspended in 50 μl of antibody buffer (wash buffer containing 0.01% Digitonin and 2 mM EDTA) and incubated with the primary antibody at a 1:20 dilution overnight at 4°C. When RNase A treatment was needed, 30 μg of RNase A (Thermo Fisher Scientific, Catalog #EN0531) was added to the antibody buffer and incubated at 37°C for 1 h prior to primary antibody binding. The cell-bead slurry was washed twice with dig wash buffer (wash buffer containing 0.01% Digitonin), incubated with Protein A-MNase (pA-MN) produced in-house at a 1:130 dilution for 10 min at room temperature, and then washed twice more with dig wash buffer. The slurry was then incubated with dig wash buffer containing 2 mM CaCl2 for pA-MN digestion at 4°C for 2 h. To stop the digestion reaction, 33 μl of stop buffer (340 mM NaCl, 20 mM EDTA, 4 mM EGTA, 50 μg/ml glycogen, 50 μg/ml RNase A, 2 pg/ml heterologous spike-in *E. coli* DNA) was added, and the fragments were released by incubating at 37°C for 10 min. The supernatant was collected, and DNA was extracted using the CUTANA^™^ DNA Purification Kit (EpiCypher, Catalog # 14-0050) according to the supplier’s protocol. The resulting DNA was used as input for library preparation as previously described^16^.

#### DNA sequencing data processing

The size distributions and molar concentration of libraries were determined using an Agilent 4200 TapeStation and libraries were mixed to achieve equal representation as desired aiming for a final concentration as recommended by the manufacturer. Paired-end 50x50 bp sequencing on the Illumina NextSeq 2000 platform was performed by the Fred Hutchinson Cancer Center Genomics Shared Resources and data were analyzed as described (https://www.protocols.io/view/cut-amp-tag-data-processing-and-analysis-tutorial-e6nvw93x7gmk/v1).

For processing sequencing data, we used cutadapt 4.4^69^ to trim adapters from 50 bp paired-end reads with parameters “-j 8 –next-seq-trim 20 -m 20 -a

AGATCGGAAGAGCACACGTCTGAACTCCAGTCA -A AGATCGGAAGAGCGTCGTGTAGGGAAAGAGTGT -Z”

We used Bowtie2 2.5.1^70^ to map the paired-end 50 bp reads to the hg19 human genome reference sequence from UCSC with parameters “–very-sensitive-local –soft-clipped-unmapped-tlen –dovetail –no-mixed –no-discordant -q –phred33 -I 10 -X 1000”. Spike-in *E. coli* reads were mapped to Ensembl masked R64-1-1 with parameters “ –end-to-end –very-sensitive –no-overlap –no-dovetail –no-mixed –no-discordant -q –phred33 -I 10 -X 1000”

#### Data analysis and visualization

We used bedtools^71^ genomecov to generate bedGraph files, which were then converted to bigWig format using bedGraphToBigWig. Coverage-normalized bigWig files represent the fraction of counts at each base pair, scaled by the size of the reference hg19 genome (3,095,693,983), ensuring that if the counts were uniformly distributed, each position would have a value of 1. This scaling helps identify enriched protein epitope signals on DNA. Raw count bigWig files, containing unnormalized counts, were used to evaluate drug-induced RNAPIIS5P, RNAPIIS2P and H3K36me3 changes across the genome, with CUT&Tag libraries pooled at equal volumes for comparable input. Spike-in calibrated BigWig files were generated to assess drug-induced genome-wide changes in U2AF2 CUT&RUN signal. Calibration was performed using a scale factor calculated by dividing 10,000 by the number of mapped *E. coli* fragments. Genomic bigWig tracks were displayed using Integrated Genome Viewer (IGV). Heatmaps were generated with deepTools (version 3.5.1)^72^ using computeMatrix and plotHeatmap. For k-means clustering analysis on 12,397 intron-containing genes we used the plotHeatmap function in deepTools with –kmeans 3 option on U2AF2 CUT&RUN data.

Total U2AF2 counts on 1,765 U2AF2-high genes in control and RNase A-treated samples were calculated using bedtools intersect with the -a -b -c option. Differential enrichment analysis was then performed using DESeq2 (v.1.32.0) to determine if U2AF2 counts significantly changed on some genes upon RNase A treatment^73^. Gene Ontology (GO) enrichment analysis was performed using the clusterProfiler package in R Studio (v.4.1.1)^74^ with the enrichGO function. The input gene list was analyzed against the org.Hs. eg.db database for all ontologies (Biological Process, Cellular Component, and Molecular Function) by setting ont = “ALL”. P-values were adjusted using the Benjamini-Hochberg method (pAdjustMethod = “BH”), and the top 10 GO terms with an adjusted p-value below 0.01 (qvalueCutoff = 0.01) were identified as significant and visualized using the dotplot function in R. Information about the gene lists categorized in this study—including intron-containing, intronless, U2AF2 chromatin-bound, RNase A-sensitive, U2AF2-high active, U2AF2-low active, inactive, splicing factor genes, and those with exon inclusion errors—is provided in Data S1. Information about the exons and introns categorized in this study—including all exons and introns of U2AF2-high active and U2AF2-low active genes, as well as those with splicing errors upon U2AF2 KD—is provided in Data S2.

The CUT&RUN data for NPAT (Gene Expression Omnibus: GSM3609774, GSM3391666),^18,68^ MYC (GSM2433146), MAX (GSM2433145), and CTCF (GSM2433138) were previously published by our lab^16,18,68^ and have been reprocessed into coverage-normalized bigWig files for this study. The processed U2AF2 ChIP-seq bigWig file (ENCODE: ENCFF220HWQ),^24^ representing the fold change of IP signal over input control, was downloaded from the ENCODE database. K562 hg19-mapped and processed bigWig files for the minus and plus strands of U2AF2 eCLIP signal^42^ (ENCFF573ZFK and ENCFF996ZIG) and their input control signal (ENCFF498CTW and ENCFF780WFJ) were also obtained from the ENCODE database. These BigWig files were converted to bedGraph format using bigWigToBedGraph. The resulting bedGraph files were then processed into final bed files, which were used to measure U2AF2 eCLIP and input total fragment counts on genes, exons, or introns using bedtools intersect with the -a -b -c option. RNA-seq data mapped to hg19 from the chromatin fraction (ENCSR000CPY), including the minus and plus strand bigWig tracks (ENCFF778XHU and ENCFF690JHR) and a TSV file containing TPM values representing nascent transcript levels (ENCFF746OZI), were downloaded from the ENCODE database. Total RNA-seq data from K562 control (ENCSR344XID) and U2AF2 KD (ENCSR904CJQ) cells were downloaded from ENCODE, including the minus and plus strand bigWig tracks for control cells (ENCFF651UUW and ENCFF383SGN) and U2AF2 KD cells (ENCFF365VKX and ENCFF074AMW). The PSI values (referred to as IncLevelDifference) and p-values (referred to as PValue) for K562 control and U2AF2 KD, used to identify splicing errors in exon inclusion, intron removal, 5’ splice sites, and 3’ splice sites upon U2AF2 KD, were obtained from a tar file downloaded from ENCODE (ENCFF468ZZD). Total RNA-seq data from HepG2 control (ENCSR104ABF) and U2AF2 KD (ENCSR426UUG) cells were downloaded from ENCODE. The PSI and p-values for HepG2 control and U2AF2 KD were downloaded from ENCODE (ENCFF930SEB). The two replicates of MNase-seq FASTQ files (GSM7761912, GSM7761914) in K562 cells were downloaded from SRA^43^ and remapped to the hg19 genome to generate BED files containing all fragment information, which were then merged into a single BED file. From this BED file, only nucleosome-sized fragments (≥150 bp) were selected using awk ‘$NF >= 150’. The number of these fragments was divided either by the total gene length in kilobases or by the total GC numberin kilobases, for nucleosome occupancy calculations on genes. The published RPKM values (referred to as RPKM_4sUchr1), co-transcriptional splicing efficiency (referred to as splicing_index_4sUchr1) of 4sU-labeled chromatin-associated transcripts, and 5’ and 3’ splice site strengths (referred to as X5SS_score and X3SS_score) in K562 cells were obtained from Table S5 in Drexler et al.^5^ The published SON TSA-seq scores (referred to as tsa_value) in K562 cells were obtained from Table S3 in Chen et al.^23^

Other graphs, including boxplots, violin plots, scatter plots, bar plots, and pie charts, were created using base R graphics packages in R Studio. The box in boxplots represents the interquartile range (25^th^ to 75^th^ percentile), with a horizontal line inside the box denoting the median. The lower and upper whiskers extend to the smallest and largest values within 1.5 times the interquartile range, respectively.

### QUANTIFICATION AND STATISTICAL ANALYSIS

For each CUT&Tag or CUT&RUN experiment, at least two biological replicates were included. Statistical comparisons between groups for U2AF2 eCLIP IP/input fold change, U2AF2 occupancy, RNAPIIS2P occupancy, TPM, RPKM, gene length, exon density, % GC content, and nucleosome occupancy were performed using the Mann-Whitney-Wilcoxon test in R Studio (v.4.1.1). Bonferroni correction was applied to adjust for multiple comparisons and only adjusted p-values below the corrected threshold of 0.01 were considered statistically significant.

## Supplementary Material

Data S1

Data S2

3

Supplemental information can be found online at https://doi.org/10.1016/j.molcel.2025.04.013.

## Figures and Tables

**Table:** KEY RESOURCES TABLE

REAGENT or RESOURCE	SOURCE	IDENTIFIER
Antibodies		
Guinea Pig anti-Rabbit IgG (Heavy & Light Chain)	Antibodies Online	Cat#ABIN101961; RRID: AB_10775589
Rabbit monoclonal anti-H3K36me3	Epicypher	Cat#13-0058; RRID: AB_3665058
Rabbit monoclonal anti-RNAPIIS2P	Cell Signaling Technology	Cat#13499S; RRID: AB_2798238
Rabbit monoclonal anti-RNAPIIS5P	Cell Signaling Technology	Cat#13523S; RRID: AB_2798246
Rabbit monoclonal anti-SF3B1	Cell Signaling Technology	Cat#14434S; RRID: AB_2798479
Rabbit polyclonal anti-U2AF35	Bethyl Laboratories	Cat#A302-079A; RRID: AB_1604295
Rabbit polyclonal anti-U2AF65	Abcam	Cat#ab37530; RRID: AB_883336
Rabbit polyclonal anti-SRSF1	Thermo Fisher Scientific	Cat# A302-052A, RRID: AB_1604258
Rabbit polyclonal anti-SRSF3	MBL International	Cat# RN080PW, RRID: AB_11160964
Chemicals, peptides, and recombinant proteins		
Triptolide	Selleckchem	Cat#S3604
Flavopiridol hydrochloride hydrate	Sigma-Aldrich	Cat#FL3055
Actinomycin D	Sigma-Aldrich	Cat#A9415
EPZ-719	MedChem Express	Cat#HY-139626
pAG-Tn5	Epicypher	Cat# 15-1117
Roche Complete EDTA free protease inhibitor tablet	Sigma-Aldrich	Cat# 05056489001
Bio-Mag Plus Concanavalin A	Bangs Laboratories	Cat#BP531
Thermolabile Proteinase K	New England Biolabs	Cat#P8111S
RNase A	Thermo Fisher Scientific	Cat#EN0531
Critical commercial assays		
KAPA PCR Master Mix	Roche	Cat#07958846001
CUTANA^™^ DNA Purification Kit	Epicypher	Cat#14-0050
HighPrep PCR Cleanup System	MagBio	Cat#AC-60500
High Sensitivity D1000 TapeStation system	Agilent	Cat#5067-5584
Deposited data		
CUT&RUN of splicing factors, RNAPIIS2P	This study	GEO: GSE270327
CUT&Tag of H3K36me3, RNAPIIS2P, RNAPIIS5P	This study	GEO: GSE270327
CUT&RUN of NPAT	Meers et al.^68^	GEO: GSM3609774
CUT&RUN of NPAT in K562 cells	Janssens et al.^18^	GEO: GSM3391666
CUT&RUN of MAX, MYC, CTCF in K562 cells	Skene et al.^16^	GEO: GSE84474
U2AF2 ChIP-seq in K562 cells	ENCODE Project Consortium.^24^	ENCODE: ENCFF220HWQ
U2AF2 eCLIP in K562 cells	Nostrand et al.^42^	ENCODE: ENCSR893RAV
RNA-seq from the chromatin fraction of K562	ENCODE Project Consortium^24^	ENCODE: ENCSR000CPY
Total RNA-seq data from K562 control	Nostrand et al.^42^	ENCODE: ENCSR344XID
Total RNA-seq data from K562 U2AF2 KD	Nostrand et al.^42^	ENCODE: ENCSR904CJQ
MNase-seq in K562 cells	Zumer et al.^43^	GEO: GSM7761912; GSM7761914
Total RNA-seq data from HepG2 control	ENCODE Project Consortium^24^	ENCODE: ENCSR104ABF
Total RNA-seq data from HepG2 U2AF2 KD	ENCODE Project Consortium.^24^	ENCODE: ENCSR426UUG
SON TSA-seq in K562 cells	Chen et al.^23^	GEO: GSE66019
Experimental models: Cell lines		
K562	ATCC	Cat# CCL-243; RRID: CVCL_0004
Software and algorithms		
Cutadapt 4.4	Martin^69^	https://github.com/marcelm/cutadapt/
Bowtie2 2.5.1	Langmead and Salzberg^70^	https://github.com/BenLangmead/bowtie2/releases/tag/v2.5.1
bedtools (v.2.30.0)	Quinlan and Hall^71^	https://packages.guix.gnu.org/packages/bedtools/2.30.0/
deepTools (version 3.5.1)	Ramírez et al.^72^	https://deeptools.readthedocs.io/en/latest
DESeq2 (v.1.32.0)	Love et al.^73^	https://github.com/mikelove/DESeq2
clusterProfiler R package (v.4.10.1)	Wu et al.^74^	https://github.com/YuLab-SMU/clusterProfiler
R studio V4.1.1	R-project	https://www.r-project.org/

## Data Availability

• All primary sequencing data have been deposited as paired-end fastq files in the Gene Expression Omnibus under accession code GEO: GSE270327. This study also analyzed existing, publicly available CUT&RUN,^16,18,68^ U2AF2 ChIP-seq,^24^ U2AF2 eCLIP,^42^ MNase-seq,^43^ SON TSA-seq,^23^ and RNA-seq^24,42^ data. Accession numbers for all datasets are listed in the key resources table. • This paper does not report original code. • Any additional information required to reanalyze the data reported in this paper is available from the lead contact upon request.

## Highlights

- Splicing factor U2AF2 transfers from RNAPII to chromatin via the H3K36me3 mark
- U2AF2 then transfers to splice junctions on nascent RNA
- U2AF2 enhances exon inclusion in its targeted genes
- U2AF2 improves splicing accuracy in splicing factor transcripts
