# Supplementary material for: Chromatin-bound U2AF2 splicing factor ensures exon inclusion
Source: Mol Cell. Author manuscript; Available in PMC 2026 Apr 13. (PMC13075997; doi:10.1016/j.molcel.2025.04.013)
Supplement: 3 [file NIHMS2148059-supplement-3.pdf]

Molecular Cell, Volume *85*

Supplemental information

Chromatin-bound U2AF2 splicing factor ensures exon inclusion

Weifang Wu, Kami Ahmad, and Steven Henikoff

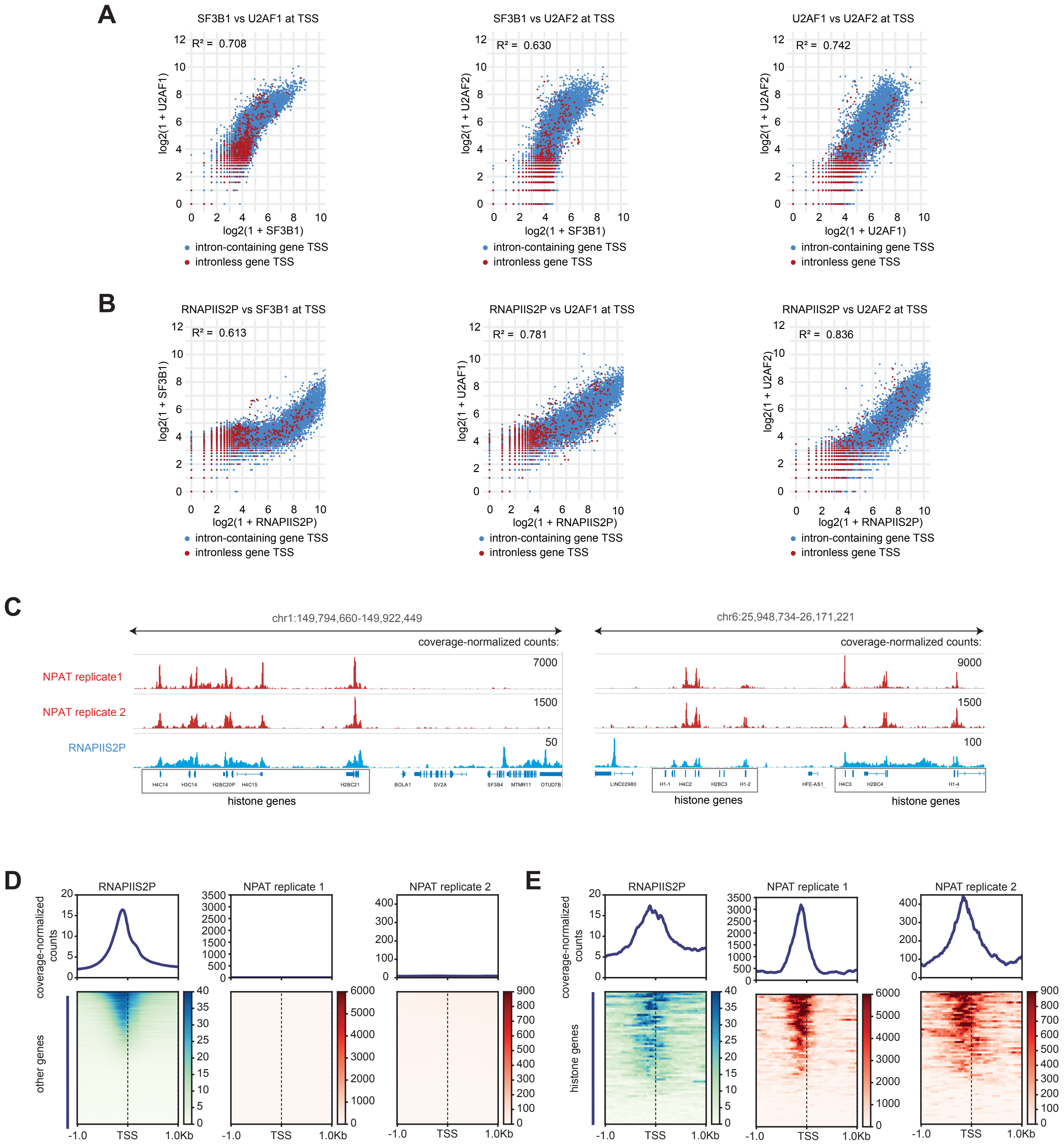
A
SF3B1 vs U2AF1 at TSS
R² = 0.708
log2(1 + U2AF1)
log2(1 + SF3B1)
SF3B1 vs U2AF2 at TSS
R² = 0.630
log2(1 + U2AF2)
log2(1 + SF3B1)
U2AF1 vs U2AF2 at TSS
R² = 0.742
log2(1 + U2AF2)
log2(1 + U2AF1)
intron-containing gene TSS
intronless gene TSS
B
RNAPIIS2P vs SF3B1 at TSS
R² = 0.613
log2(1 + SF3B1)
RNAPIIS2P vs U2AF1 at TSS
R² = 0.781
log2(1 + U2AF1)
RNAPIIS2P vs U2AF2 at TSS
R² = 0.836
log2(1 + U2AF2)
log2(1 + RNAPIIS2P)
intron-containing gene TSS
intronless gene TSS
C
chr1:149,794,660-149,922,449
chr6:25,948,734-26,171,221
coverage-normalized counts:
NPAT replicate1
NPAT replicate 2
RNAPIIS2P
7000
1500
50
9000
1500
100
H4C14
H3C14
H2BC20P
H4C15
H2BC21
BOLA1
SV2A
SF3B4
MTMR11
OTUD7B
LINC02980
H1-1
H4C2
H2BC3
H1-2
HFE-AS1
H4C3
H2BC4
H1-4
histone genes
D
E
RNAPIIS2P
NPAT replicate 1
NPAT replicate 2
coverage-normalized counts
other genes
histone genes
-1.0
TSS
1.0Kb

Figure S1

Figure S1 Positive correlation between RNAPIIS2P and splicing factors at promoters, related to Figure 1

(**A**) Scatterplots showing positive correlations between log2-transformed fragment counts of SF3B1 and U2AF1 (left), SF3B1 and U2AF2 (middle), and U2AF1 and U2AF2 (right) within 2 kb of TSSs in intron-containing (blue) and intronless (red) genes. (**B**) Scatterplots showing positive correlations between log2-transformed fragment counts of RNAPIIS2P and SF3B1 (left), RNAPIIS2P and U2AF1 (middle), and RNAPIIS2P and U2AF2 (right) within 2 kb of TSSs in intron-containing (blue) and intronless (red) genes. Each dot represents one gene TSS. (**C**) Browser tracks showing NPAT and RNAPIIS2P CUT&RUN signals over histone gene clusters and nearby regions. The y-axis shows coverage-normalized counts. (**D, E**) Heatmaps (bottom) and average plots (top), aligned to TSSs of other genes (**D**) or histone genes (**E**), showing coverage-normalized counts for RNAPIIS2P and NPAT.

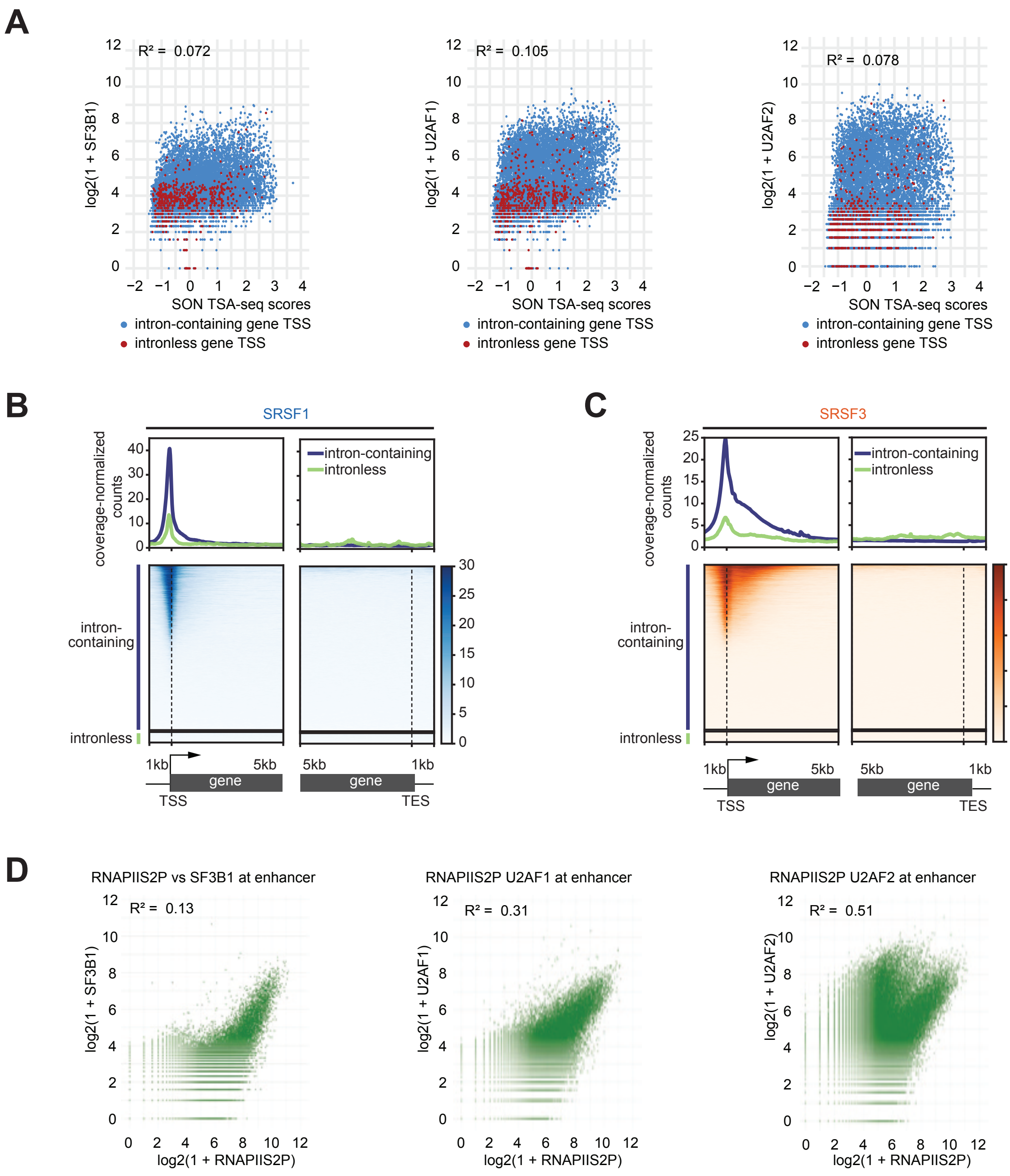
A
R² = 0.072
R² = 0.105
R² = 0.078
log2(1 + SF3B1)
log2(1 + U2AF1)
log2(1 + U2AF2)
SON TSA-seq scores
intron-containing gene TSS
intronless gene TSS
B
SRSF1
C
SRSF3
coverage-normalized counts
intron-containing
intronless
intron-containing
intronless
1kb
5kb
gene
TSS
TES
D
RNAPIIS2P vs SF3B1 at enhancer
RNAPIIS2P U2AF1 at enhancer
RNAPIIS2P U2AF2 at enhancer
R² = 0.13
R² = 0.31
R² = 0.51
log2(1 + RNAPIIS2P)

Figure S2

Figure S2 Nuclear speckle localization is not required for splicing factors to bind at promoters, related to Figure 1

(**A**) Scatterplots showing the relationship between gene distance to nuclear speckles and log2-transformed fragment counts of SF3B1 (left), U2AF1 (middle), and U2AF2 (right) within 2 kb of TSSs in intron-containing (blue) and intronless (red) genes. Higher SON TSA-seq scores represent closer proximity to speckles[23]. Each dot represents one gene TSS. (**B, C**) Heatmaps (bottom) and average plots (top), aligned to the TSS or TES of 12,397 intron-containing and 793 intronless genes, showing coverage-normalized counts for SRSF1 (**B**) and SRSF3 (**C**). (**D**) Scatterplots showing positive correlations between log2-transformed fragment counts of RNAPIIS2P and SF3B1 (left), RNAPIIS2P and U2AF1 (middle), and RNAPIIS2P and U2AF2 (right) at enhancers. The analysis included 83,870 proximal and 323,717 distal ENCODE-annotated enhancers in humans. Each dot represents one enhancer.

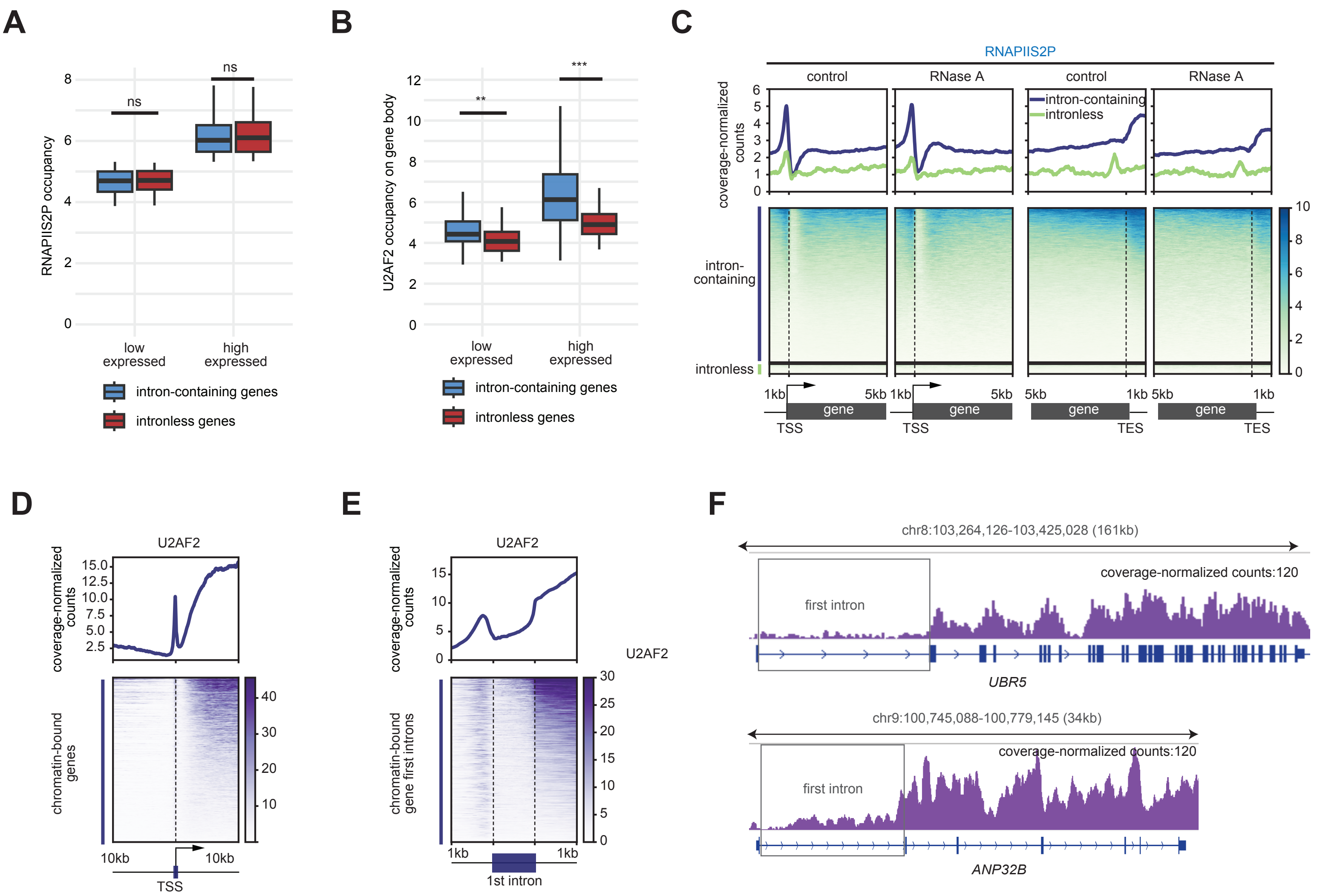

A
RNAPIIS2P occupancy
ns
ns
low expressed
high expressed
intron-containing genes
intronless genes
B
U2AF2 occupancy on gene body
**
***
low expressed
high expressed
intron-containing genes
intronless genes
C
RNAPIIS2P
control
RNase A
control
RNase A
coverage-normalized counts
intron-containing
intronless
intron-containing
intronless
1kb
5kb
1kb
5kb
5kb
1kb
5kb
1kb
gene
TSS
TES
D
U2AF2
coverage-normalized counts
chromatin-bound genes
10kb
10kb
TSS
E
U2AF2
coverage-normalized counts
chromatin-bound gene first introns
U2AF2
1kb
1kb
1st intron
F
chr8:103,264,126-103,425,028 (161kb)
first intron
coverage-normalized counts:120
UBR5
chr9:100,745,088-100,779,145 (34kb)
first intron
coverage-normalized counts:120
ANP32B

Figure S3

Figure S3 U2AF2 accumulates on gene bodies from second exons, related to Figure 2
(**A**) Boxplots showing RNAPIIS2P occupancy across whole genes, from 1 kb upstream of the TSS to 1 kb downstream of the TES, calculated as log2(1 + RNAPIIS2P RPK) for each gene group. (**B**) Boxplots showing U2AF2 occupancy across gene bodies, from 1 kb downstream of the TSS to the TES, calculated as log2(1 + U2AF2 RPK), for each gene group. Mann-Whitney-Wilcoxon test with Bonferroni correction was used. Adjusted p-value: <0.01 *, <0.001 **, <0.0001 ***. (**C**) Heatmaps (bottom) and average plots (top) aligned to the TSS or TES of intron-containing and intronless genes, displaying coverage-normalized counts for RNAPIIS2P under control or RNase A treatment conditions. (**D**) Heatmap (bottom) and average plot (top) of U2AF2 coverage-normalized counts aligned to a 10kb window around the TSS of 1,735 U2AF2 chromatin-bound genes. (**E**) Heatmap (bottom) and average plot (top) of U2AF2 coverage-normalized counts aligned to a 1kb window around the scaled first introns of chromatin-bound genes. (**F**) Browser track showing U2AF2 signals on the gene bodies beginning from the second exon. The y-axis displays coverage-normalized counts.

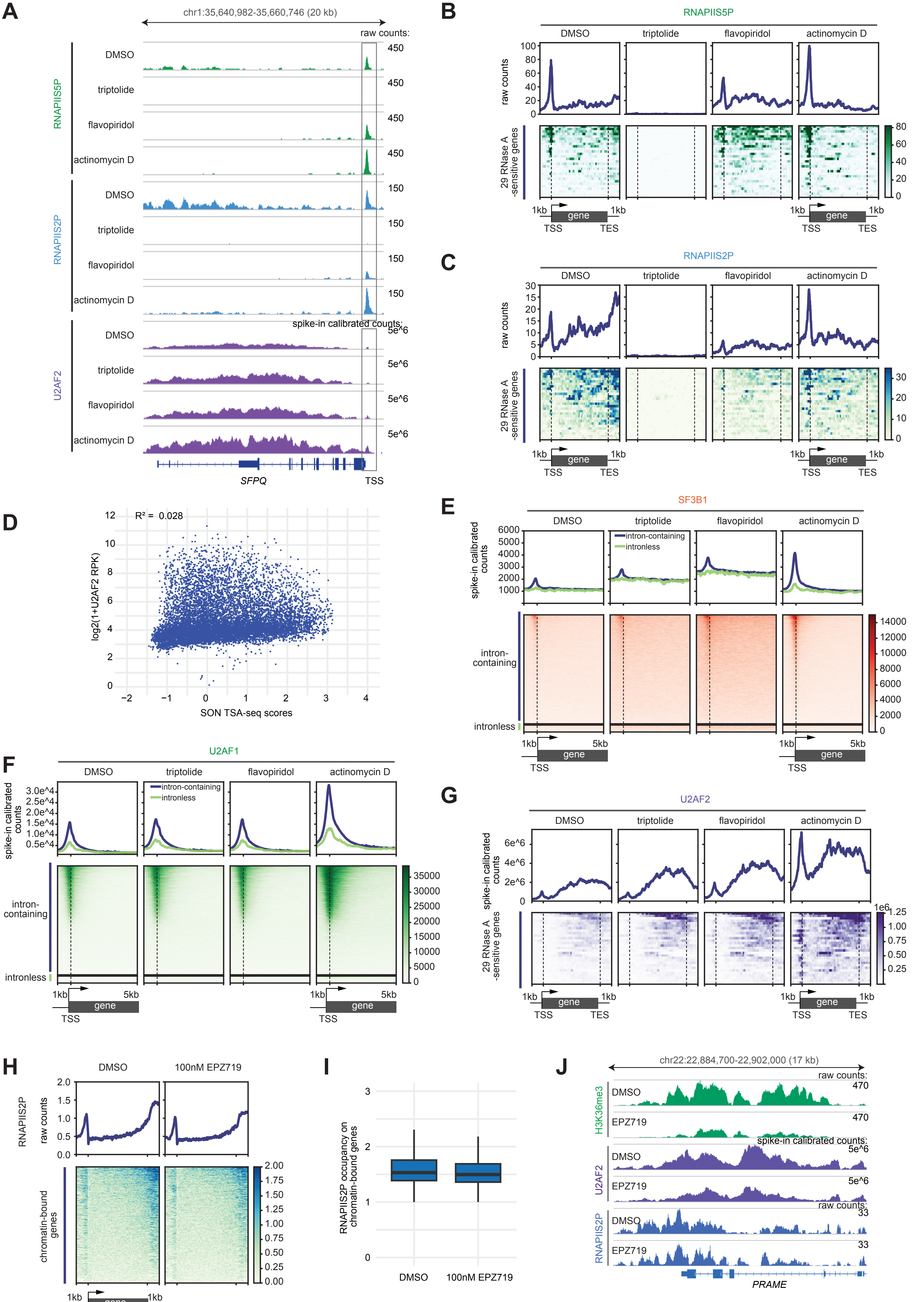

A
chr1:35,640,982-35,660,746 (20 kb)
raw counts:
RNAPIIS5P
RNAPIIS2P
U2AF2
DMSO
triptolide
flavopiridol
actinomycin D
450
150
spike-in calibrated counts:
5e^6
SFPQ
TSS
B
RNAPIIS5P
raw counts
29 RNase A -sensitive genes
1kb
gene
TSS
TES
C
RNAPIIS2P
D
R² = 0.028
log2(1+U2AF2 RPK)
SON TSA-seq scores
E
SF3B1
spike-in calibrated counts
intron-containing
intronless
5kb
F
U2AF1
G
U2AF2
1e6
H
DMSO
100nM EPZ719
chromatin-bound genes
I
RNAPIIS2P occupancy on chromatin-bound genes
J
chr22:22,884,700-22,902,000 (17 kb)
H3K36me3
EPZ719
PRAME

Figure S4

Figure S4 RNAPII is not required to maintain U2AF2, related to Figure 3

(**A**) Browser track showing the distribution of RNAPIIS5P, RNAPIIS2P and U2AF2 on the RNase A-sensitive gene *SFPQ* under the indicated treatment conditions. The y-axis shows raw counts for RNAPIIS5P and RNAPIIS2P, and spike-in calibrated counts for U2AF2. (**B, C**) Heatmaps (bottom) and average plots (top) showing raw RNAPIIS5P (**B**) and RNAPIIS2P (**C**) counts on RNase A-sensitive genes under the indicated treatments. (**D**) Scatterplots showing the relationship between nuclear speckle proximity and U2AF2 occupancy on gene bodies. The published SON TSA-seq scores were compared with U2AF2 occupancy in K562 cells. Each dot represents a single intron-containing gene. (**E, F**) Heatmaps (bottom) and average plots (top) aligned to the TSS of 12,397 intron-containing and 793 intronless genes, showing spike-in calibrated counts for SF3B1 (**E**) and U2AF1 (**F**) under the indicated treatments. (**G**) Heatmaps (bottom) and average plots (top) showing the spike-in calibrated counts for U2AF2 on RNase A-sensitive genes under the indicated treatment conditions. (**H**) Heatmaps (bottom) and average plots (top) showing raw RNAPIIS2P counts on U2AF2 chromatin-bound genes under the indicated treatments. (**I**) Boxplot of raw RNAPIIS2P counts on these genes. (**J**) Browser track snapshot of H3K36me3, U2AF2 and RNAPIIS2P distribution on the *PRAME*. The y-axis shows raw counts for H3K36me3 and RNAPIIS2P, and spike-in calibrated counts for U2AF2.

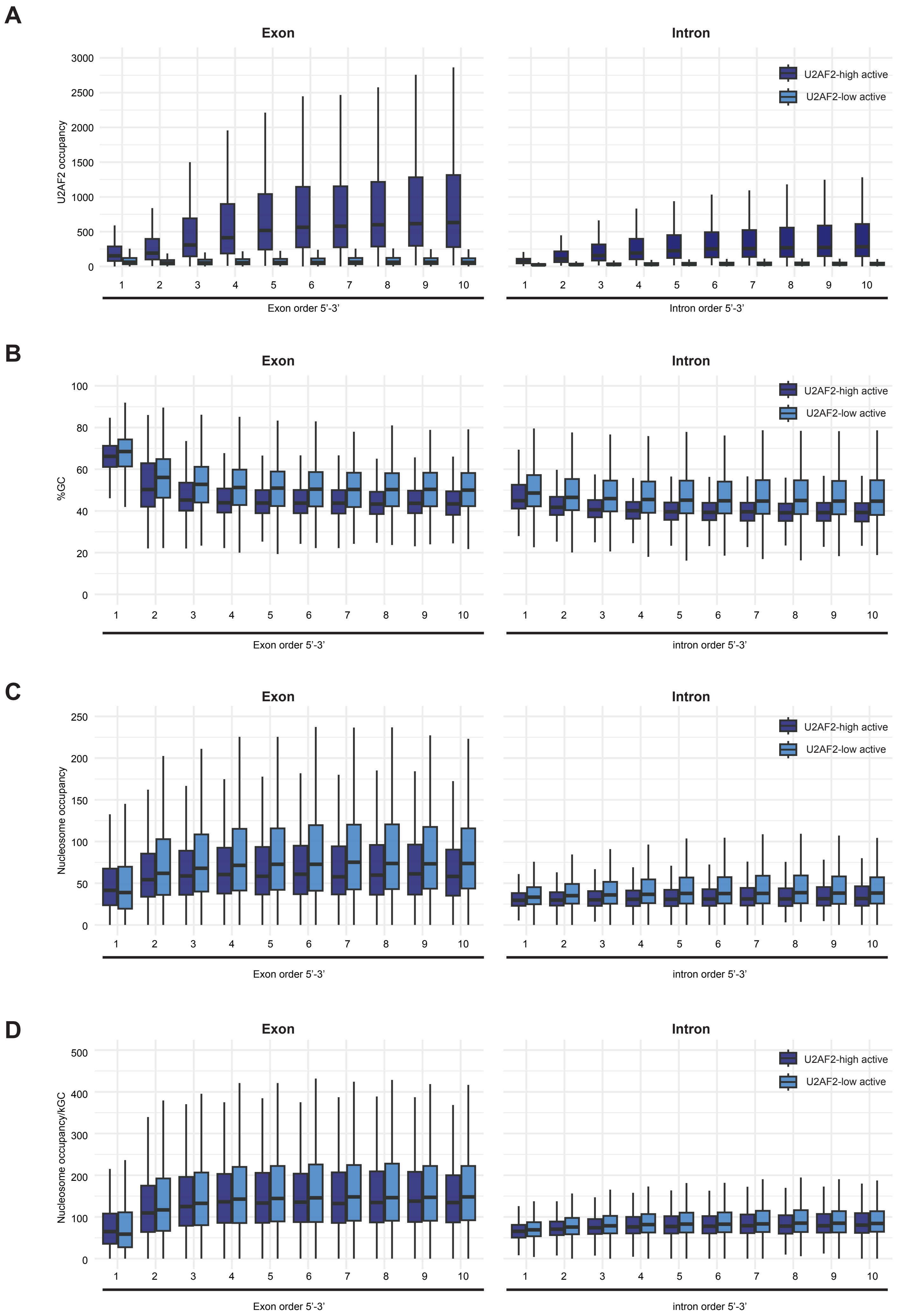
A
Exon
Intron
U2AF2 occupancy
U2AF2-high active
U2AF2-low active
Exon order 5'-3'
Intron order 5'-3'
B
Exon
Intron
%GC
U2AF2-high active
U2AF2-low active
Exon order 5'-3'
intron order 5'-3'
C
Exon
Intron
Nucleosome occupancy
U2AF2-high active
U2AF2-low active
Exon order 5'-3'
intron order 5'-3'
D
Exon
Intron
Nucleosome occupancy/kGC
U2AF2-high active
U2AF2-low active
Exon order 5'-3'
intron order 5'-3'

Figure S5

Figure S5 U2AF2 preferentially binds to exons, related to Figure 4
(**A-D**) The levels of %GC (**A**), Nucleosome occupancy (**B**), Nucleosome occupancy/kGC (**C**), and U2AF2 occupancy (U2AF2 RPK) (**D**) on the first 10 exons and introns in U2AF2-high active and U2AF2-low active genes. Nucleosome occupancy = total number of nucleosomal fragment (>=150bp) on regions (exon or intron) / (region length in kilobases). Nucleosome occupancy/kGC = total number of nucleosomal fragment (>=150bp) on regions (exon or intron) / total number of GC in kilobases.

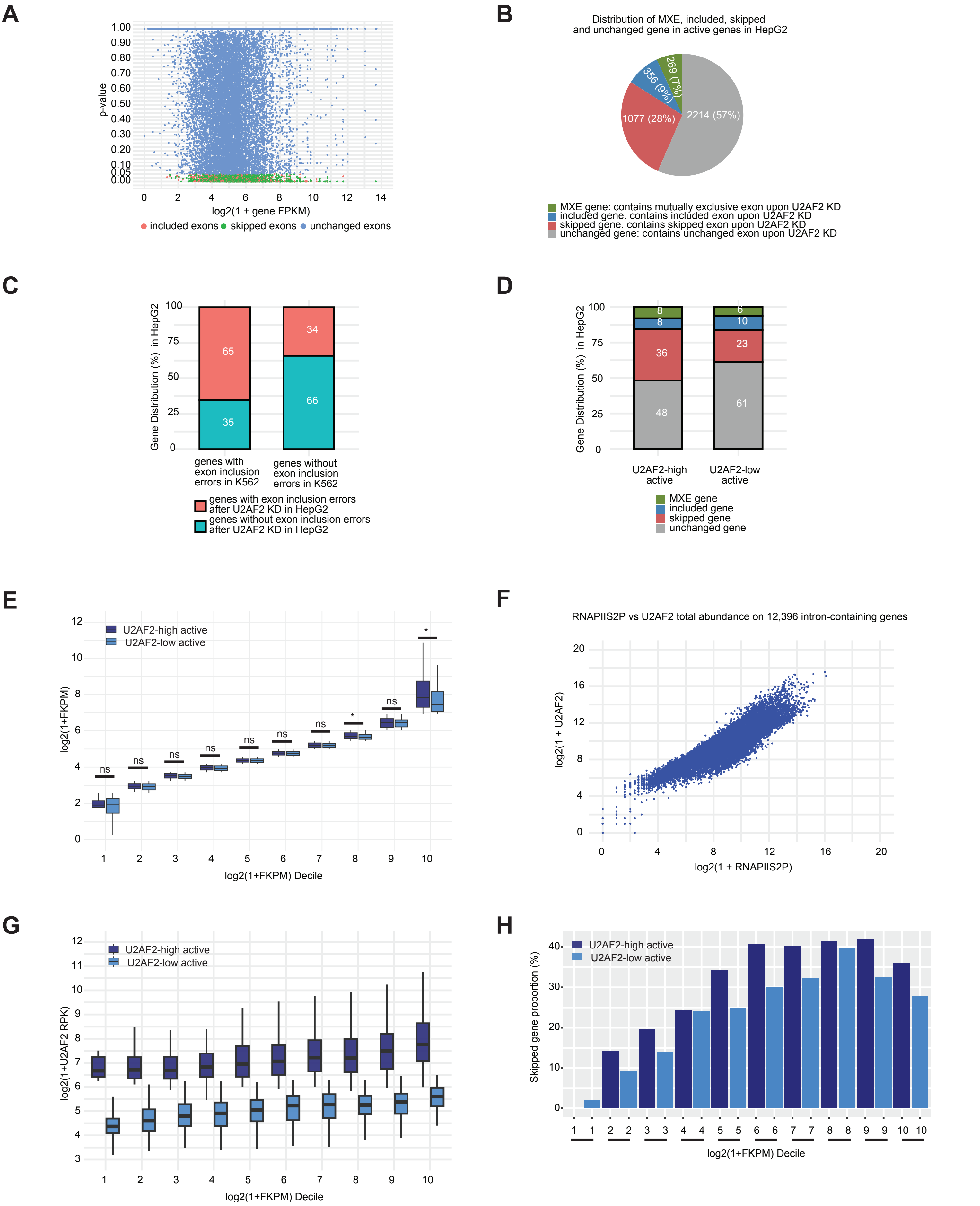

A
p-value
log2(1 + gene FPKM)
included exons
skipped exons
unchanged exons
B
Distribution of MXE, included, skipped and unchanged gene in active genes in HepG2
269 (7%)
356 (9%)
1077 (28%)
2214 (57%)
MXE gene: contains mutually exclusive exon upon U2AF2 KD
included gene: contains included exon upon U2AF2 KD
skipped gene: contains skipped exon upon U2AF2 KD
unchanged gene: contains unchanged exon upon U2AF2 KD
C
Gene Distribution (%) in HepG2
genes with exon inclusion errors in K562
genes without exon inclusion errors in K562
genes with exon inclusion errors after U2AF2 KD in HepG2
genes without exon inclusion errors after U2AF2 KD in HepG2
D
Gene Distribution (%) in HepG2
U2AF2-high active
U2AF2-low active
MXE gene
included gene
skipped gene
unchanged gene
E
U2AF2-high active
U2AF2-low active
log2(1+FKPM)
log2(1+FKPM) Decile
F
RNAPIIS2P vs U2AF2 total abundance on 12,396 intron-containing genes
log2(1 + U2AF2)
log2(1 + RNAPIIS2P)
G
U2AF2-high active
U2AF2-low active
log2(1+U2AF2 RPK)
log2(1+FKPM) Decile
H
U2AF2-high active
U2AF2-low active
Skipped gene proportion (%)
log2(1+FKPM) Decile

Figure S6

Figure S6 Chromatin-bound U2AF2 enhances exon inclusion, related to Figure 5

(**A**) Scatterplots showing the relationship between log_2-transformed gene FPKM and p-value for exon PSI between WT and U2AF2 KD K562 cells. (**B**) Pie chart showing the distribution of unchanged, skipped, included, and MXE genes in the same active gene group from K562 in HepG2. (**C**) Distribution of genes with or without exon inclusion errors in HepG2 after U2AF2 KD, compared to K562. (**D**) The percentage of unchanged genes, skipped genes, included genes, and MXE genes in the same U2AF2-high active and U2AF2-low active gene groups categized from K562 in HepG2. **(E)** Boxplots showing log_2-transformed FPKM (Fragments Per Kilobase of transcript per Million mapped reads) for U2AF2-high and U2AF2-low active gene classes across 10 deciles of log_2-transformed FPKM in WT cells. FPKM values are obtained from published data[42]. Mann-Whitney-Wilcoxon test with Bonferroni correction was used. Adjusted p-value: <0.01 *, <0.001 **, <0.0001 ***. (**F**) Scatterplots showing positive correlation between total abundance of RNAPIIS2P and U2AF2 on intron-containing genes. Total abundance of RNAPIIS2P or U2AF2 on each intron-containing gene is calculated as log_2-transformed total mapped fragment number of RNAPIIS2P or U2AF2 on regions including 1 kb upstream and downstream of the gene / (gene length in kilobases). (**G**) Boxplots showing the distribution of U2AF2 occupancy on genes. (**H**) The percentage of genes with skipped exons upon U2AF2 KD in U2AF2-high active and U2AF2-low active gene groups across 10 deciles of log_2-transformed FPKM values.

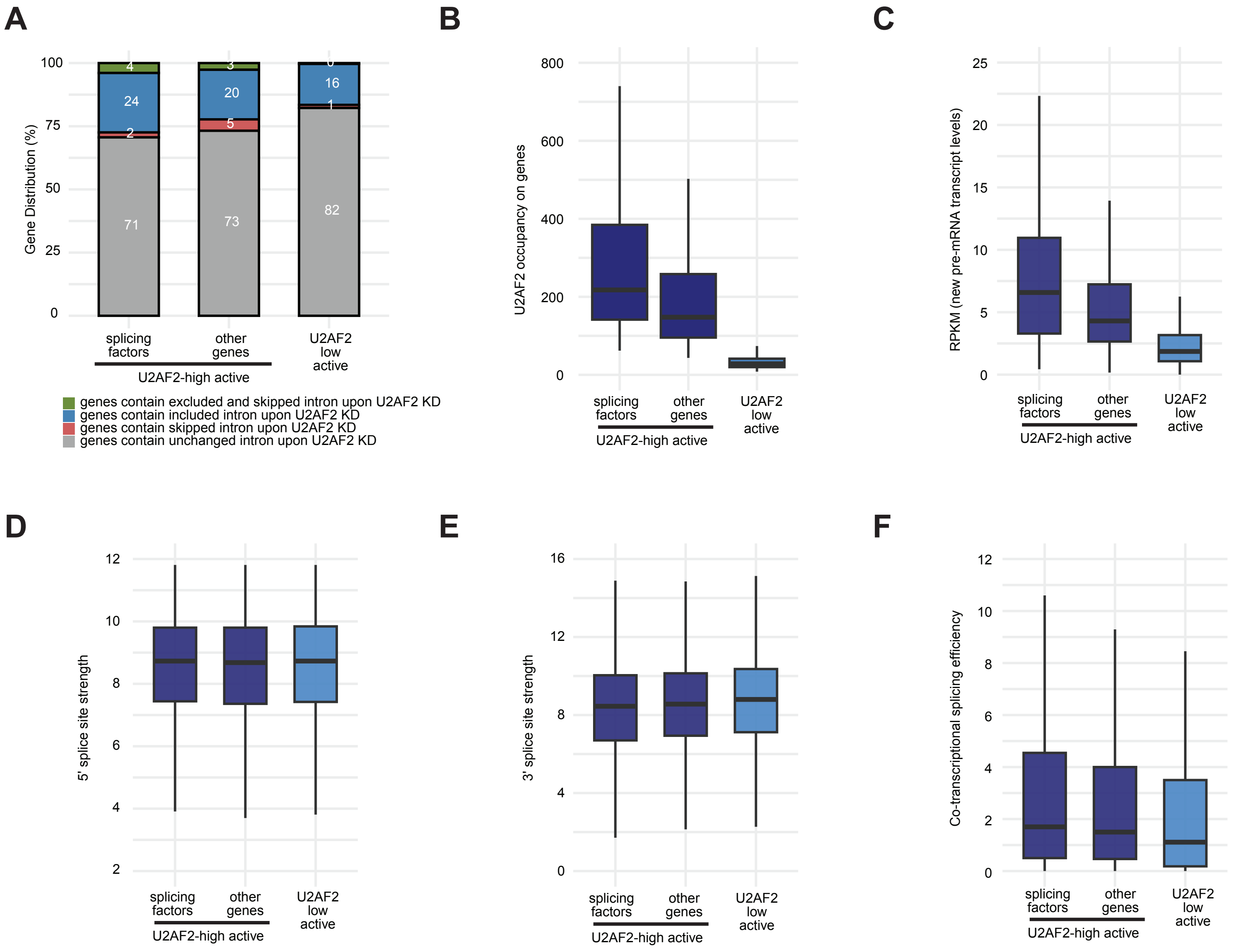

A
B
C
D
E
F
Gene Distribution (%)
100
75
50
25
0
4
24
2
71
3
20
5
73
0
16
1
82
splicing factors
other genes
U2AF2 low active
U2AF2-high active
genes contain excluded and skipped intron upon U2AF2 KD
genes contain included intron upon U2AF2 KD
genes contain skipped intron upon U2AF2 KD
genes contain unchanged intron upon U2AF2 KD
U2AF2 occupancy on genes
RPKM (new pre-mRNA transcript levels)
5' splice site strength
3' splice site strength
Co-transcriptional splicing efficiency

Figure S7

Figure S7 Chromatin-bound U2AF2 enhances splicing efficiency in splicing factor genes, related to Figure 6

(**A**) The percentage (%) of genes with intron removal errors in splicing factor genes, other genes and U2AF2-low active gene groups upon U2AF2 KD. (**B-F)** Boxplots showing U2AF2 occupancy (U2AF2 RPK) (**B**), new pre-mRNA transcript levels (RPKM) (**C**), 5' splice site strength (**D**), 3' splice site strength (**E**), and co-transcriptional splicing efficiency (**F**) across splicing factor genes, other genes, and U2AF2-low active genes. The values of 5' and 3' splice site strength, FPKM, co-transcriptional splicing efficiency of 4sU-labeled chromatin-associated new pre-mRNA transcripts are obtained from published data.[5] Splicing efficiency for each gene was determined by counting the number of read pairs that span exon junctions by at least 3 nucleotides, and measuring the number of spliced reads divided by unspliced reads; splicing efficiency = 2 × spliced read pairs / (5′ splice site unspliced + 3′ splice site unspliced read pairs).
